# Supplementary material for: A GPCR-based yeast biosensor for biomedical, biotechnological, and point-of-use cannabinoid determination
Source: Nat Commun. 2022 Jun 27;13:3664. doi: 10.1038/s41467-022-31357-6 (PMC9237071; doi:10.1038/s41467-022-31357-6)

Supplementary Information for

A GPCR-based yeast biosensor for biomedical, biotechnological, and point-of-use cannabinoid determination

Karel Miettinen[1], Nattawat Leelahakorn[1], Aldo Almeida[1,2], Yong Zhao[1], Lukas R. Hansen[1], Iben Egebæk Nikolajsen[1], Jens B. Andersen[3], Michael Givskov[3], Dan Staerk[4], Søren Bak[1], and Sotirios C. Kampranis[1, *]

[1]Biochemical Engineering Group, Plant Biochemistry Section, Department of Plant and Environmental Sciences, University of Copenhagen, Thorvaldsensvej 40, 1871 Frederiksberg C, Denmark.

[2]Bioremediation Laboratory, Faculty of Biological Sciences, Autonomous University of Coahuila, Carretera Torreón-Matamoros km. 7.5, Torreón, Coahuila, 27000, Mexico

[3]Department of Immunology and Microbiology, Faculty of Health and Medical Sciences, University of Copenhagen, Blegdamsvej 3B, 2200 Copenhagen, Denmark

[4]Department of Drug Design and Pharmacology, Faculty of Health and Medical Sciences, University of Copenhagen, Universitetsparken 2, 2100 Copenhagen, Denmark

*Corresponding author: Email: soka@plen.ku.dk

This PDF file includes: Supplementary Figures 1 to 19, and Supplementary Tables 1 to 11

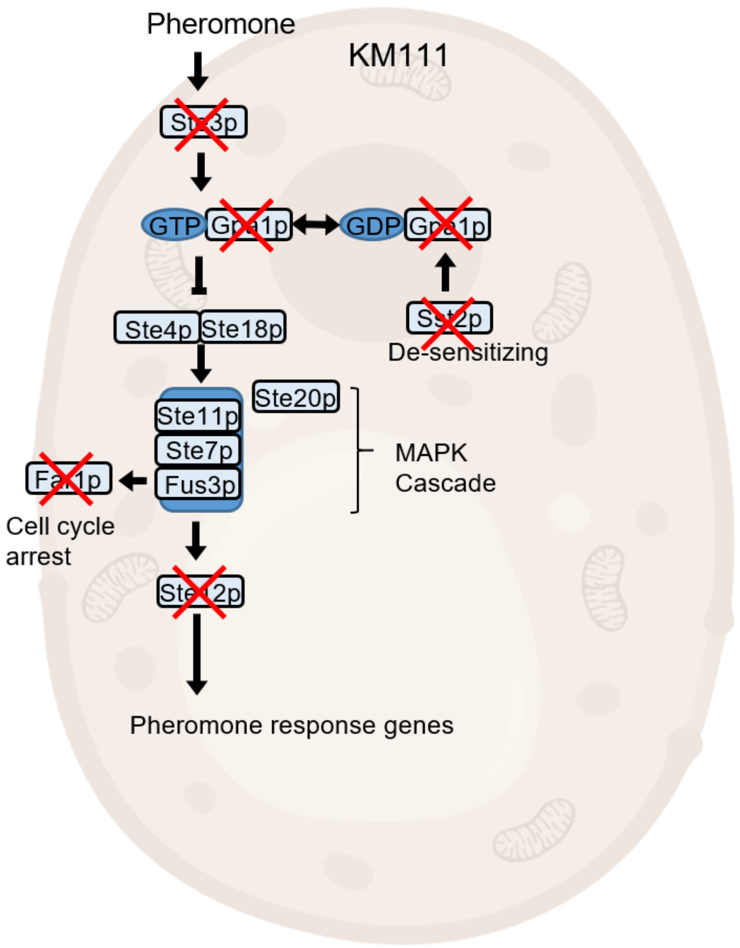

Supplementary Figure 1. Engineering the chassis strain KM111. The biosensor chassis strain KM111 was constructed by sequentially knocking out three genes (*STE3*, *STE12* and *GPA1*) that encode pheromone pathway components so that they can be replaced by custom parts. In addition, two genes (*SST2* and *FAR1*) that are detrimental to biosensor function were also deleted. First, *STE3* was knocked out by replacing it with the selection marker *URA3* by homologous recombination. Then the marker was removed using Cre/Lox recombination (LoxP sites flanking the selection marker). This approach was repeated to delete *SST2*, then *FAR1*, then *STE12*, and finally, *GPA1*.

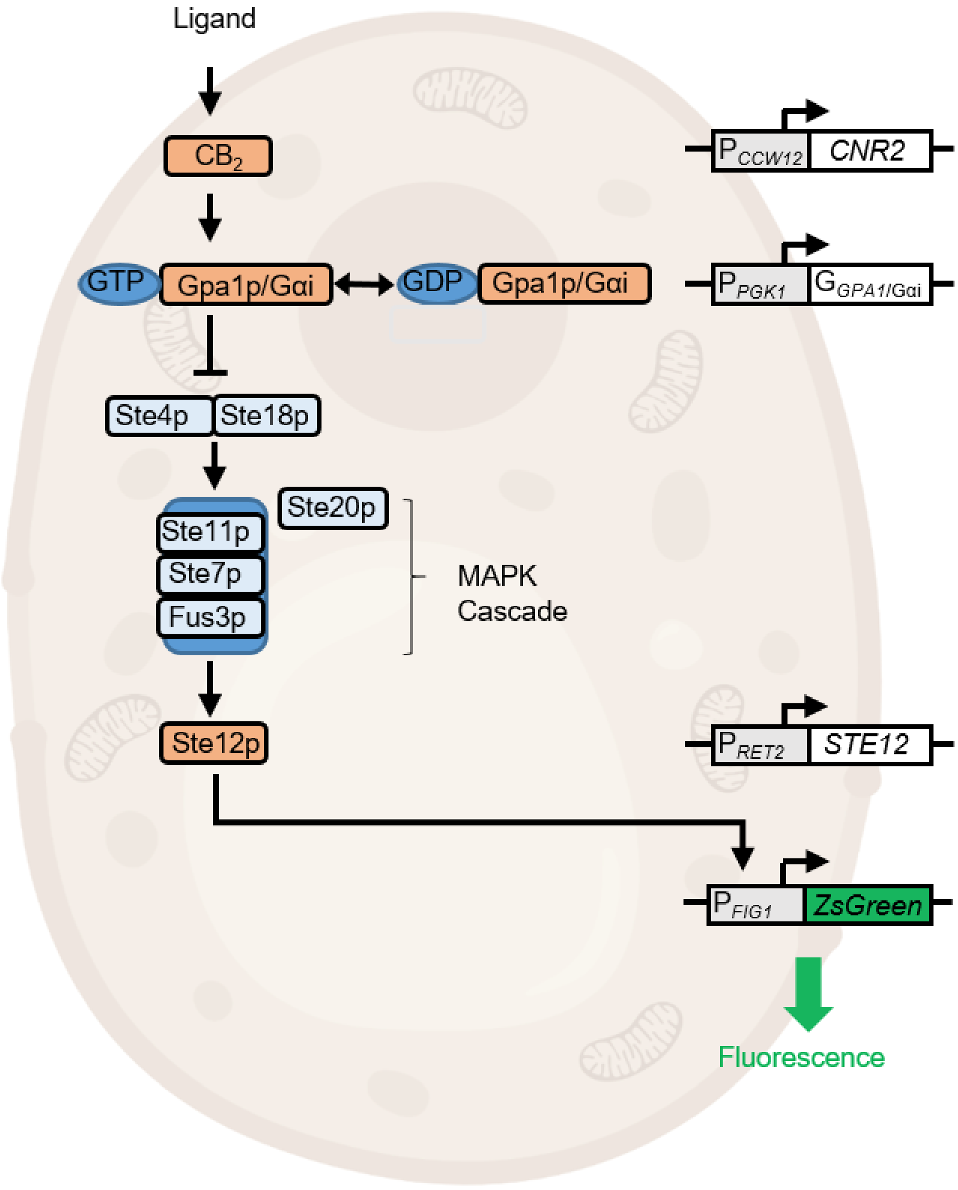

Supplementary Figure 2. The basic biosensor. The biosensor strain KM202 was constructed by integrating the *CNR2* coding sequence (gene coding for the CB_2 receptor) controlled by the P_{CCW12} promoter into the receptor module, the Gpa1/Gαi1 chimeric protein-coding sequence with the P_{PGK1} promoter into the adaptor module, *STE12* driven by the P_{RET2} promoter into the actuator module, and ZsGREEN with the P_{FIG1} promoter into the reporter module. The rest of the biosensors used in this work were constructed in a similar manner except for using different reporter or receptor module parts.

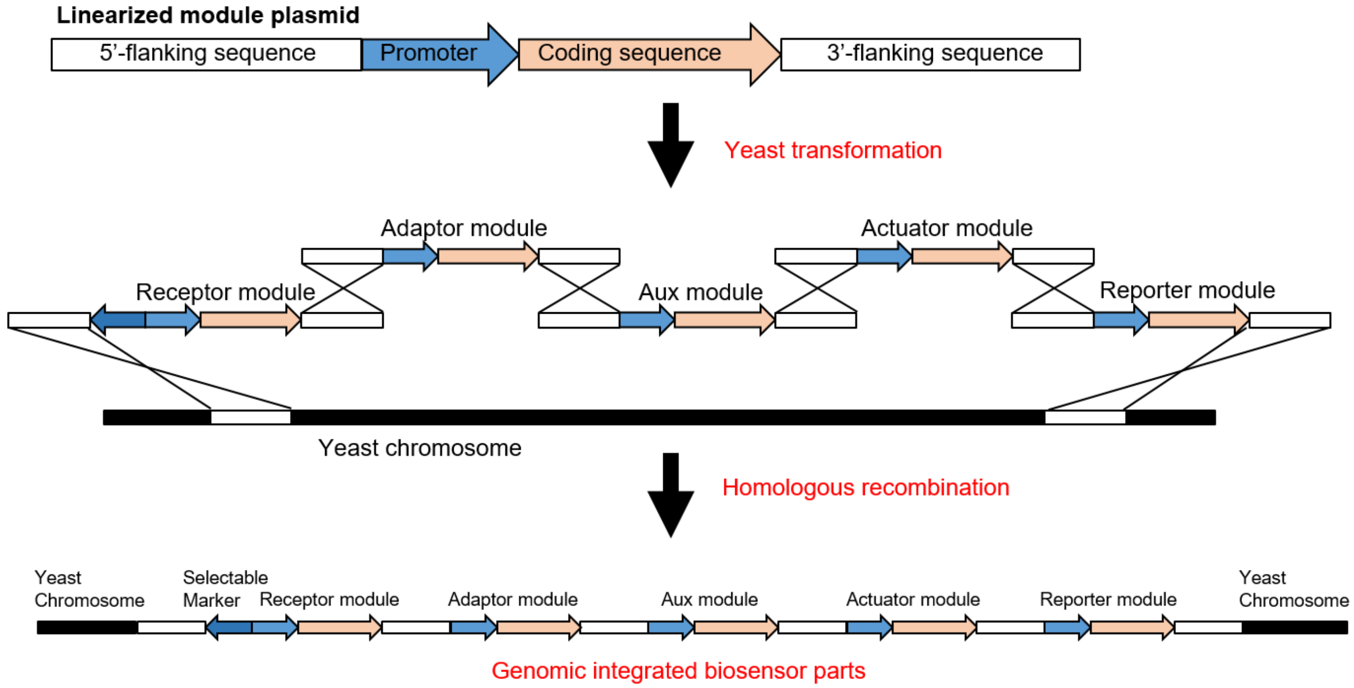

Supplementary Figure 3. Genetic integration of biosensor parts. Each biosensor was constructed by integrating parts into four modules using multipart modular genomic integration. A set of five plasmids containing a receptor part, adaptor part, actuator part, reporter part, and aux part (an empty vector that can be used for the integration of additional parts) were chosen according to the desired biosensor design. These plasmids were linearized and transformed into the chassis strain, where they were orderly integrated into the genome based on matching HR regions in each part. Integration positive colonies were selected using the *URA3* selection marker.

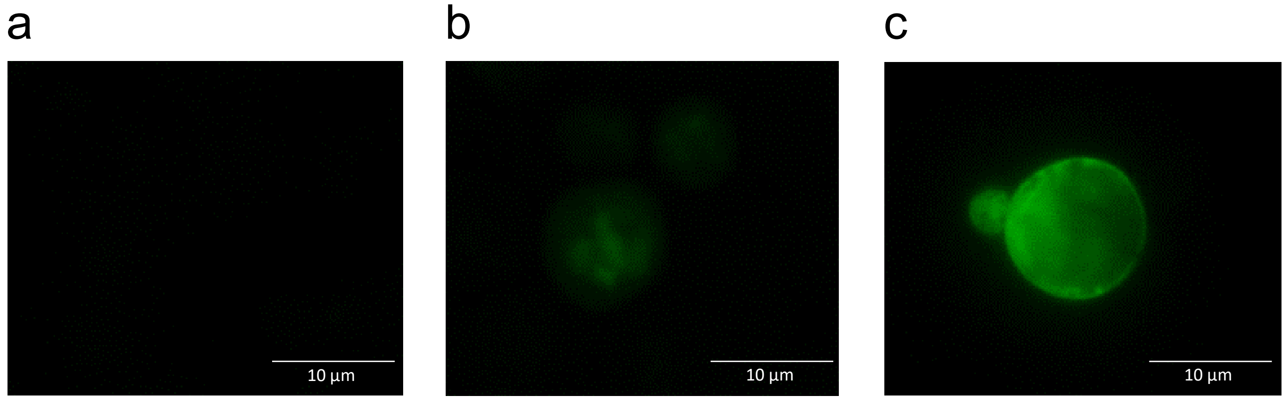

Supplementary Figure 4. Localization of the CB_2 receptor. Fluorescent microscopy analysis of yeast cells expressing a super-folder GFP-fused CB_2 receptor (CB_2-sfGFP). **(a)** Yeast cell (KM112) carrying an empty vector control (no receptor). **(b)** Example of KM113 yeast cell expressing CB_2 C-terminally fused with sfGFP. **(c)** Example of yeast cell (KM114) expressing the CB_2 receptor fused with the mating factor pre-prosequence (at the N-terminus) and sfGFP (at the C-terminus) (MFαSS-CB_2-sfGFP). These results represent single biological replicates.

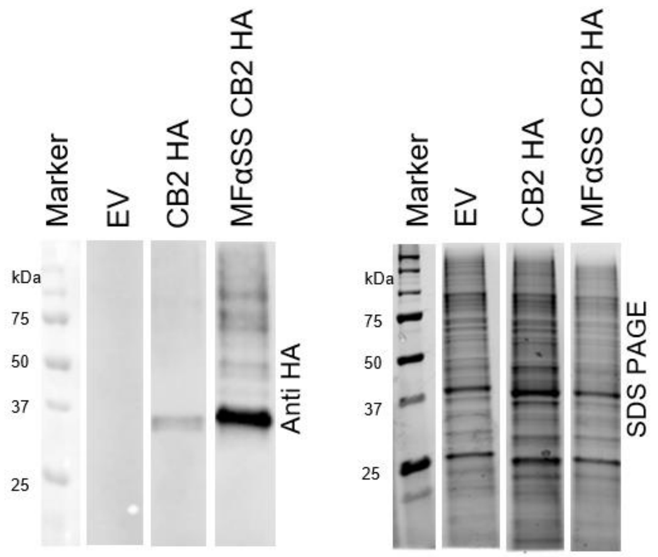

Supplementary Figure 5. CB_2 receptor accumulation in enriched yeast plasma membrane preparations. Yeast cells expressing HA-tagged CB_2 receptor (strain KM115) and mating factor-fused and HA-tagged CB_2 (strain KM206) were disrupted and membrane-rich fractions were obtained according to the procedure described in the Materials and methods section. The abundance of CB_2 receptor in the cell membranes was determined by western-blotting developed using a primary antibody for the fused HA tag (left panel). As loading control, the stain free detection of total protein from the same gel is shown on the right panel. These results represent single biological replicates.

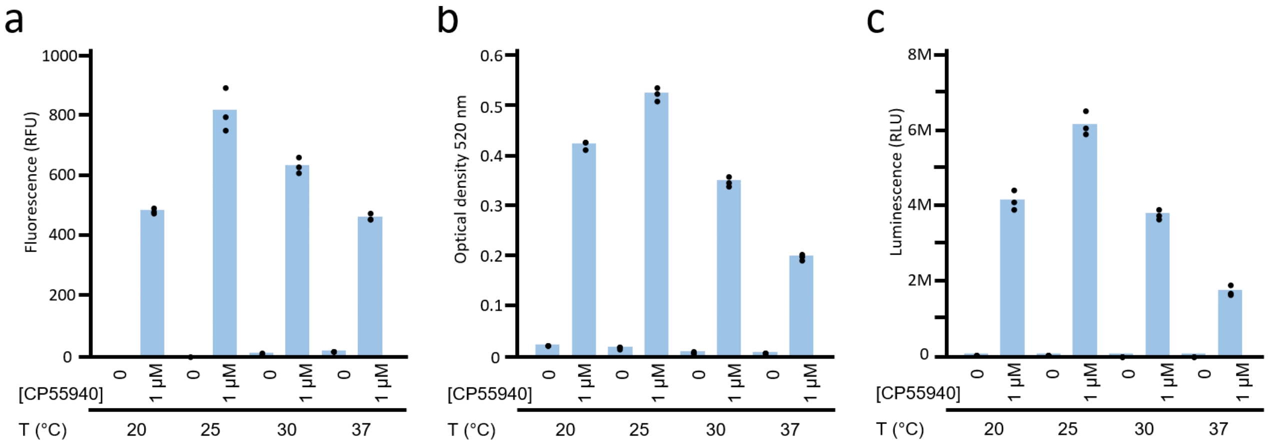

Supplementary Figure 6. Temperature dependence of biosensor performance. Each of the biosensor strains were incubated with either no cannabinoid or 1 µM CP55940 at four different temperatures 20°C, 25°C, 30°C or 37 °C. **(a)** The fluorimetric strain KM203 produced the strongest output at 25°C**. (b**) Similarly, the colorimetric strain KM205 also showed highest output at 25°C. **(c)** Likewise, the luminometric strain had the best output at 25°C. Bars correspond to means and dots to individual measurements. n=3 biologically independent samples. Source data are provided in the Source Data file.

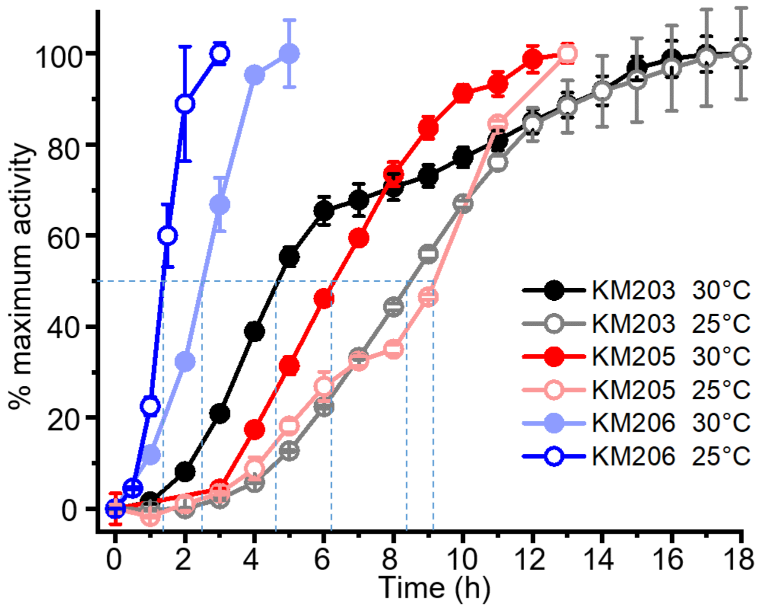

Supplementary Figure 7. Response time of the biosensor strains. In order to compare the response time of the biosensor strains KM203 (fluorescence), KM205 (betalain color with supplements) and KM206 (luminescence), all three strains were induced with 1 µM CP55940 and their outputs were measured until they reached saturation at 25°C and at 30°C. At 30°C, T_{50} (time to reach 50% of the maximum signal) was determined to be approximately 4.6 h, 6.1 h and 2.5 h for KM203, KM205 and KM206 respectively. At 25°C, T_{50} was 8.4 h, 9.1 h and 1.3 h for KM203, KM205 and KM206, respectively. Data presented as mean +/- standard deviation. n=3 biologically independent samples. Source data are provided in the Source Data file.

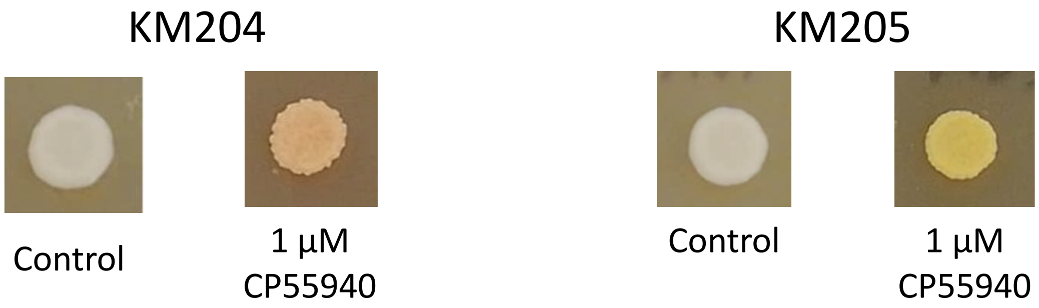

Supplementary Figure 8. Color production of the betalain reporter strains. Betalain reporter strains KM204 (betainin, red) and KM205 (betaxanthins, yellow) were grown on plates containing 1 µM CP55940. KM204 produces a clear red color and KM205 produces a yellow color when induced with the cannabinoid. In the absence of CP55940 (control), neither strain shows color build up.

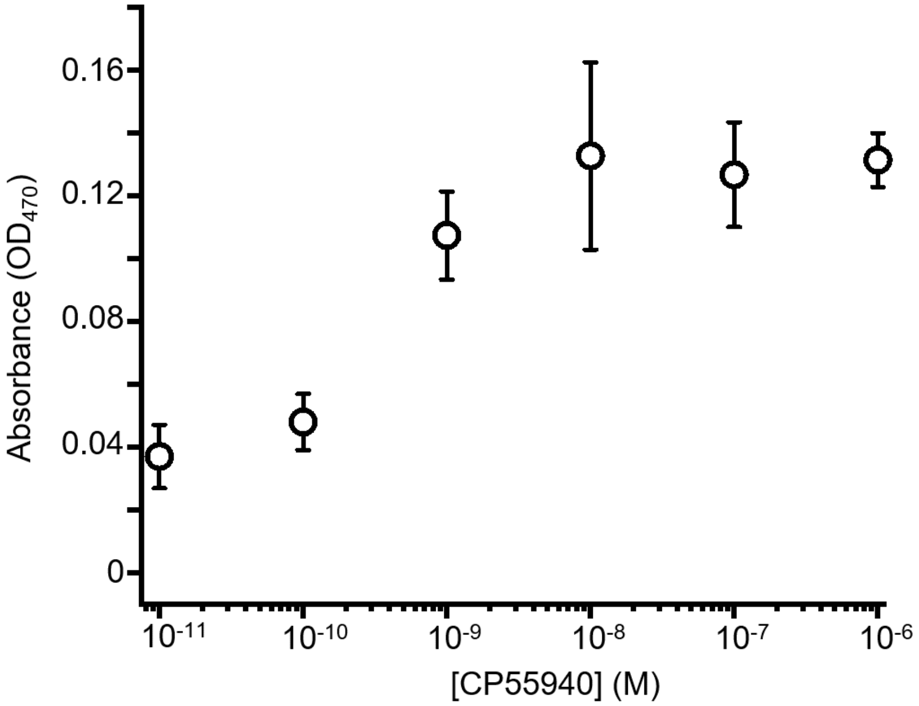

Supplementary Figure 9. CP55940 dose response of the cannabinoid biosensor strain KM205 with betaxanthin output. Incubation of the stain KM205 with a CP55940 dilution series ranging from 0 to 1 µM results in a dose dependent increase in absorbance at 470 nm (betaxanthin absorbance maximum). Data presented as mean +/- standard deviation. n=3 biologically independent samples. Source data are provided in the Source Data file.

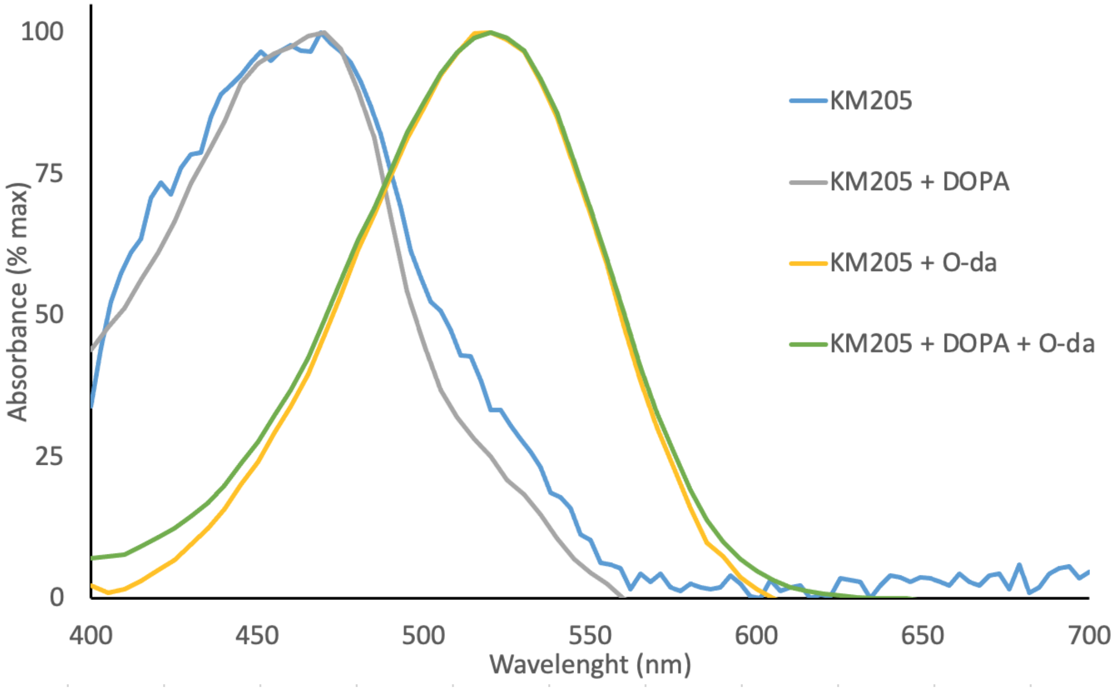

Supplementary Figure 10. Absorbance spectra of the coloured compounds produced by the betalain reporter strain KM205 supplemented with L-DOPA and or O-da. Absorbance spectrum (blue) of the supernatant of strain KM205 culture induced with 1 μM CP55940 shows the presence of betaxanthins (peak absorbance wavelength 470 nm) but no betacyanins (peak absorbance wavelength 520 nm). On the other hand, when supplemented with O-da, the spectrum (yellow) reveals the presence of betacyanins (O-da-betacyanin) but no betaxanthins. This clear distinction facilitates the use of the betalain reporter for quantitative assays. Addition of L-DOPA did not have a marked effect on the spectra (grey line, KM205 with DOPA and green line, KM205 with DOPA and O-da). These results represent single biological replicates.Source data are provided in the Source Data file.

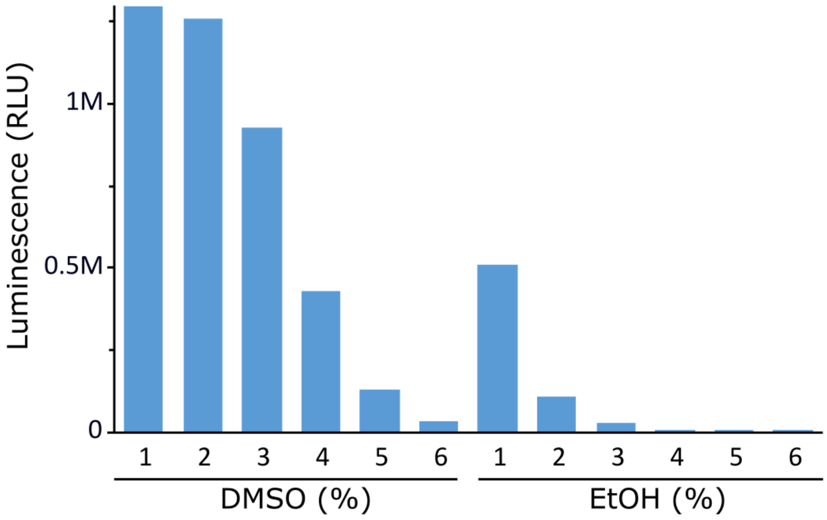

Supplementary Figure 11. Effect of solvent concentration on biosensor output. The biosensor strain KM206 with luminescence reporter was induced with 1 μM CP55940 in the presence of 1 to 6 % DMSO or ethanol. This revealed that the presence of ethanol has a negative effect on biosensor output. However, the presence of 1 or 2 % DMSO resulted in similar output levels indicating that these concentrations are not significantly detrimental to biosensor output. These results represent single biological replicates. Source data are provided in the Source Data file.

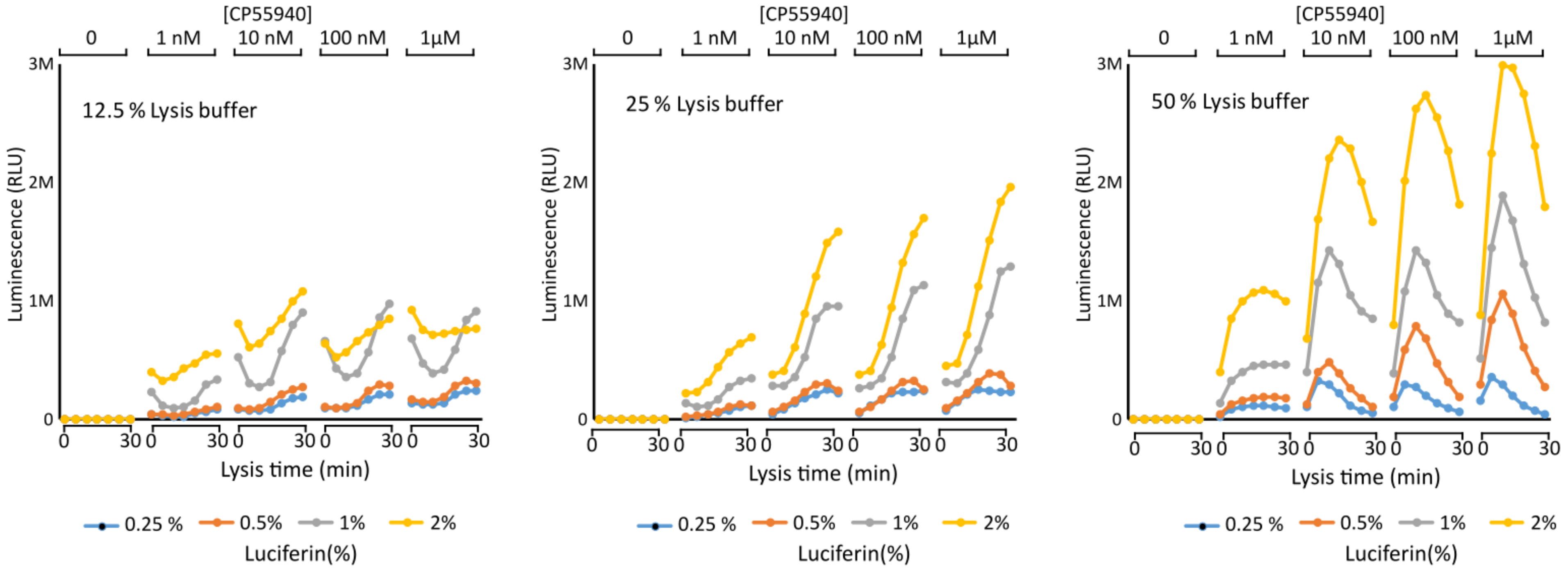

Supplementary Figure 12. Effect assay conditions on the performance of the luminescence biosensor strain. Luminescence is a dynamic process, and the output of the biosensor depends on several factors including concentration of luciferase, luciferin, and lysis buffer. In order to find the optimal parameters for our setup, we performed time series varying each parameter. The result indicated the optimal conditions for maximum output to be 2% luciferin and 50% lysis buffer for a 10-15 min incubation time. However, the biosensor showed workable output in a range of conditions, suggesting that, if required, a different set up can also be used. These results represent single biological replicates.Source data are provided in the Source Data file.

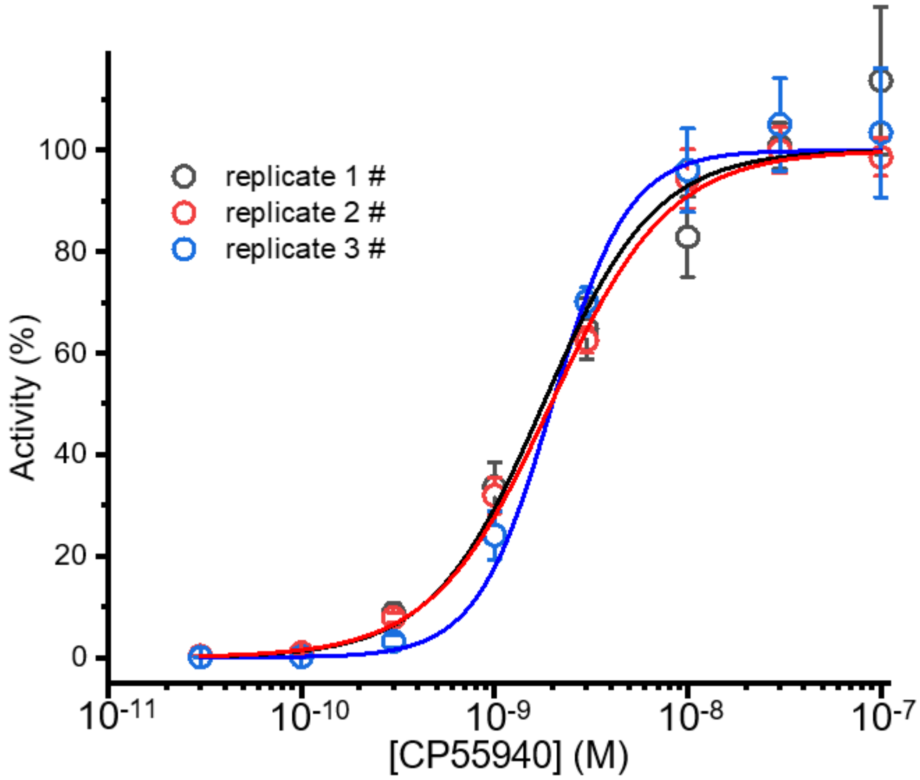

Supplementary Figure 13. Reproducibility of biosensor sensitivity. Dose-response curves of KM206 induced with CP55940 on three separate days show little variation in biosensor sensitivity (as evaluated from EC_{50}). The calculated values were 1.79 ± 0.35 nM, 1.96 ± 0.22 nM and 1.99 ± 0.21 nM indicating a mean EC_{50} of 1.93 nM and standard deviation of 0.13 nM or 5.6%. Data presented as mean +/- standard deviation. n=3 biologically independent samples. Source data are provided in the Source Data file.

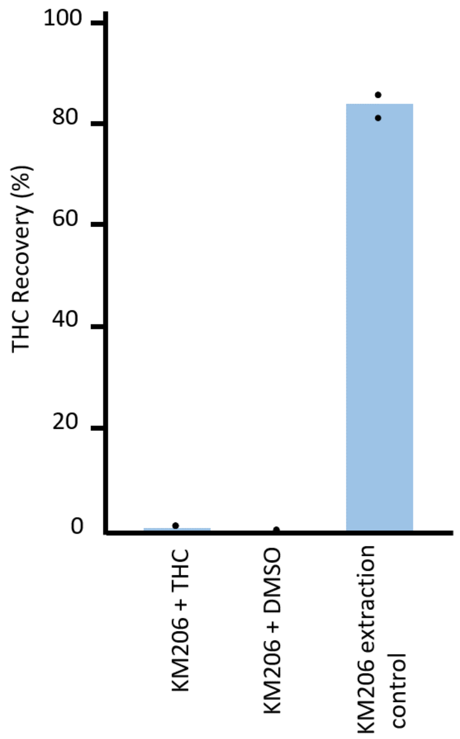

Supplementary Figure 14. Biosensor cells do not accumulate a significant portion of cannabinoids. The biosensor strains can be used to measure cannabinoid concentrations outside of the cells. To rule out that considerable amounts of the cannabinoids are absorbed into biosensor cells affecting the concentration in the media, strain KM206 was incubated for 3 h with 10 µM THC or DMSO and then the relative THC concentration in the cell pellet measured. As a control for THC extraction and recovery from the cell pellet, an equivalent (total) amount of THC was added to an equal weight cell pellet prior to extraction and sample processing, confirming that although THC can be efficiently recovered from the pellet during extraction, there is no considerable accumulation of THC within the cell or the cell membranes during the experiments. Bars correspond to means and dots to individual measurements. n=3 biologically independent samples. Source data are provided in the Source Data file.

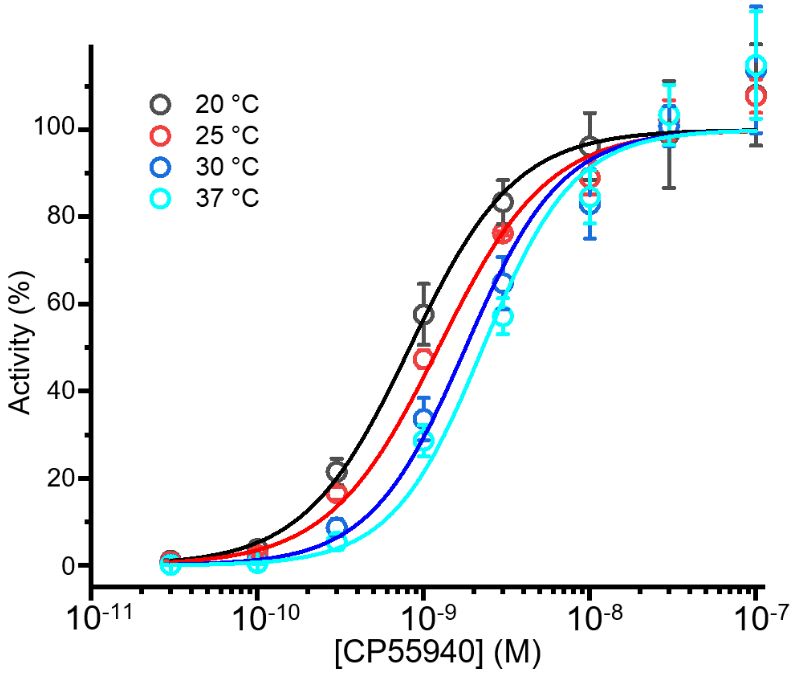

Supplementary Figure 15. Effect of temperature on biosensor sensitivity. Dose-response curves for KM206 induced with CP55940 at different temperatures shows the relation between temperature and calculated EC_{50}. These values were 0.82 ± 0.10 nM, 1.23 ± 0.19 nM, 1.79 ± 0.35 nM and 2.27 ± 0.45 nM at 20 °C, 25 °C, 30 °C and 37 °C, respectively. Data presented as mean +/- standard deviation. n=3 biologically independent samples. Source data are provided in the Source Data file.

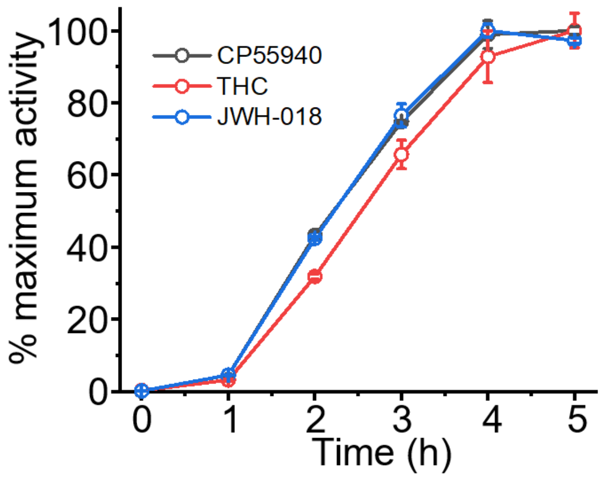

Supplementary Figure 16. Time response of the KM206 biosensor with different compounds. The biosensor strain KM206 with luminescence reporter was induced with either 1 µM CP55940 (black), 100 µM THC (red) or 100 µM JWH-018 (blue) and measurements taken once every hour. Ligand concentrations were selected to be saturating the receptor response. Data presented as mean +/- standard deviation. n=3 biologically independent samples. Source data are provided in the Source Data file.

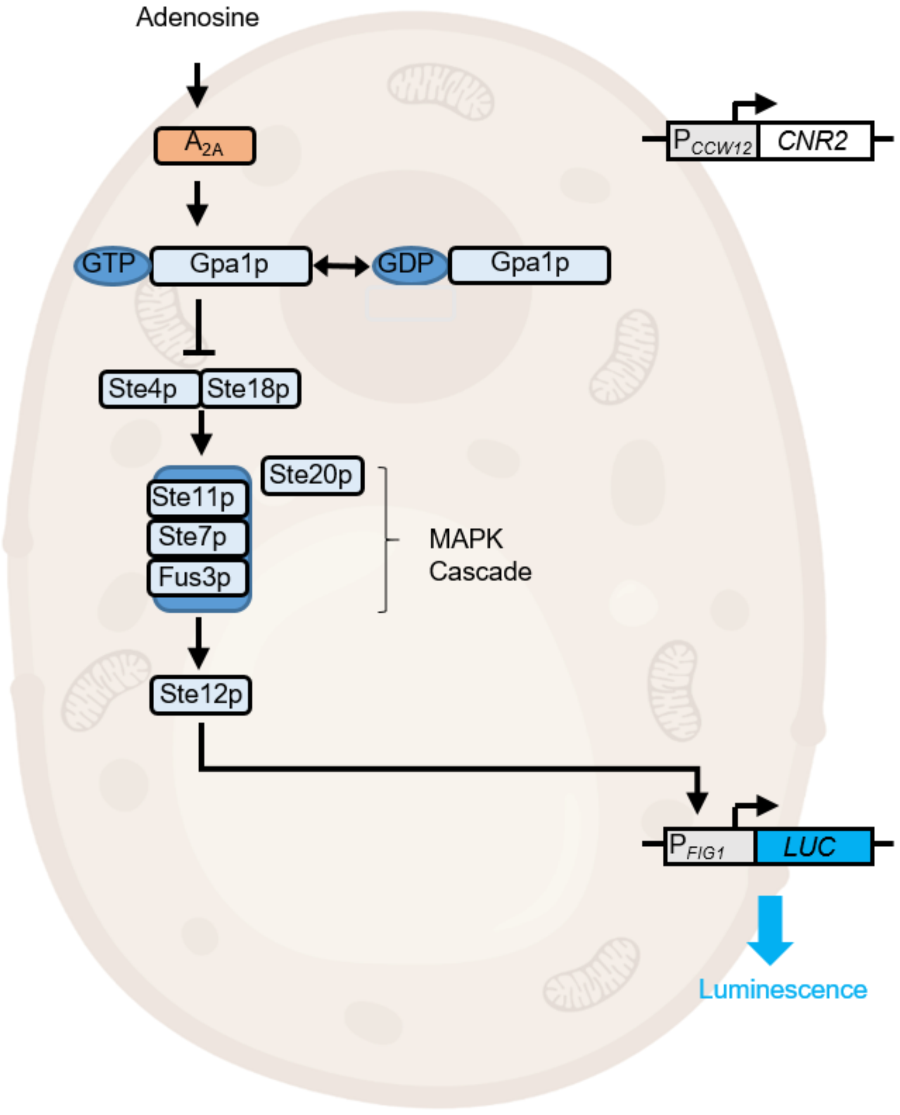

Supplementary Figure 17. The A_{2A} adenosine receptor-based biosensor for monitoring non-specific effect of compounds. This biosensor strain, KM207 was constructed to test potential non-specific inhibitory or activating effects of compounds (i.e. compounds that do not target specifically the CB_2 receptor but have a non-specific effect in the yeast cells or the rest of the engineered sensing mechanism). It was constructed by integrating the coding sequence (gene coding for the A_{2A} adenosine receptor) controlled by the P_{CCW12} promoter and the NanoLuc (nLUC) reporter into the receptor module of the chassis strain KM108. This strain has the native *STE12* and *GPA1*.

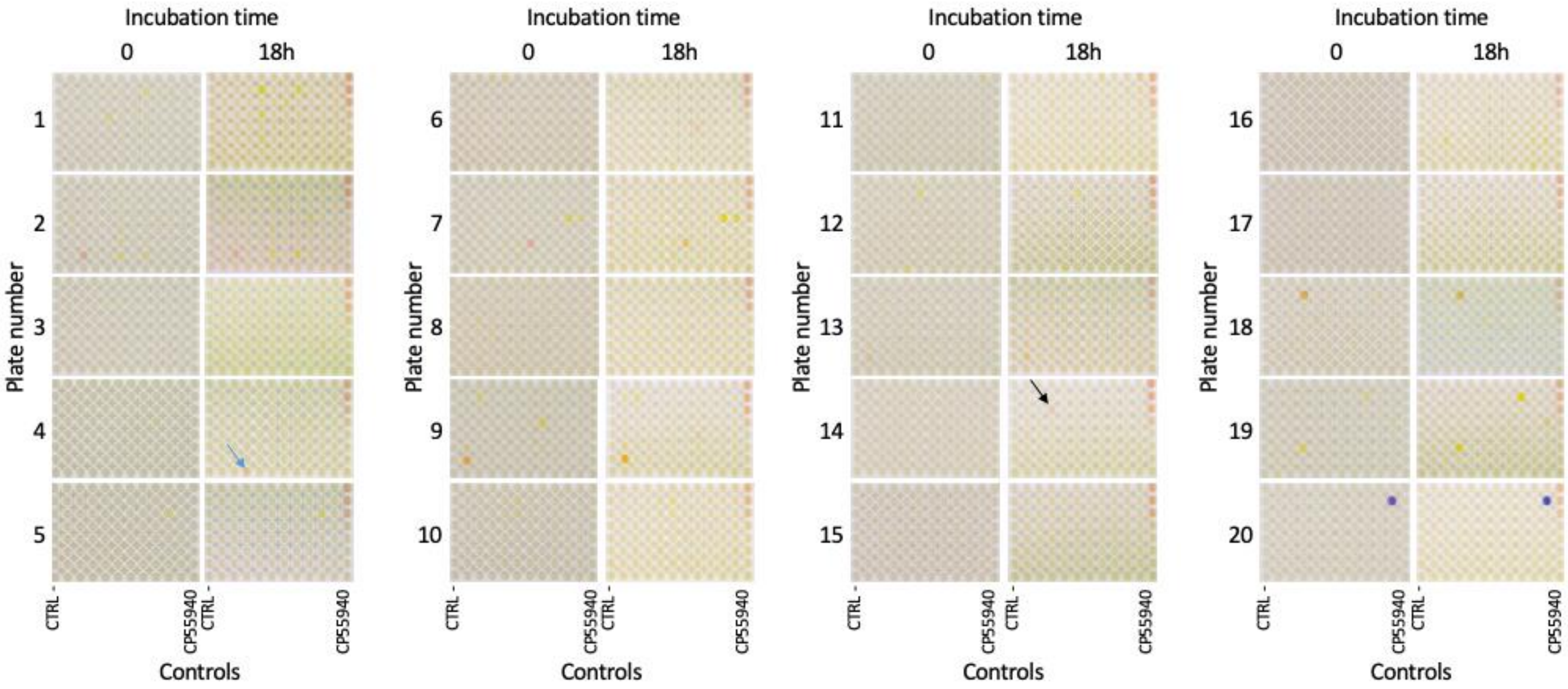

Supplementary Figure 18. High-throughput screening with the biosensor strain KM205 using the betalain reporter (O-da-betacyanin). A 100 µl-aliquot of yeast culture (OD_{600} = 5) containing 0.1 mg/mL DOPA and 0.5 mM O-da was dispensed in each well. Then, 1 µL of the chemical library was dispensed in rows 1 to 11 of each plate. Additionally, DMSO was dispensed in each well of row 1 of each plate (serving as negative control, CTRL-) and a dilution series of CP55940 (1 pM – 1 µM) to row 12 (positive control, CTRL+). The plates were incubated for 18 h at 30°C and then photographed. Two visible hits, in plate 4 blue arrow and plate 14 black arrow, appeared after the incubation.

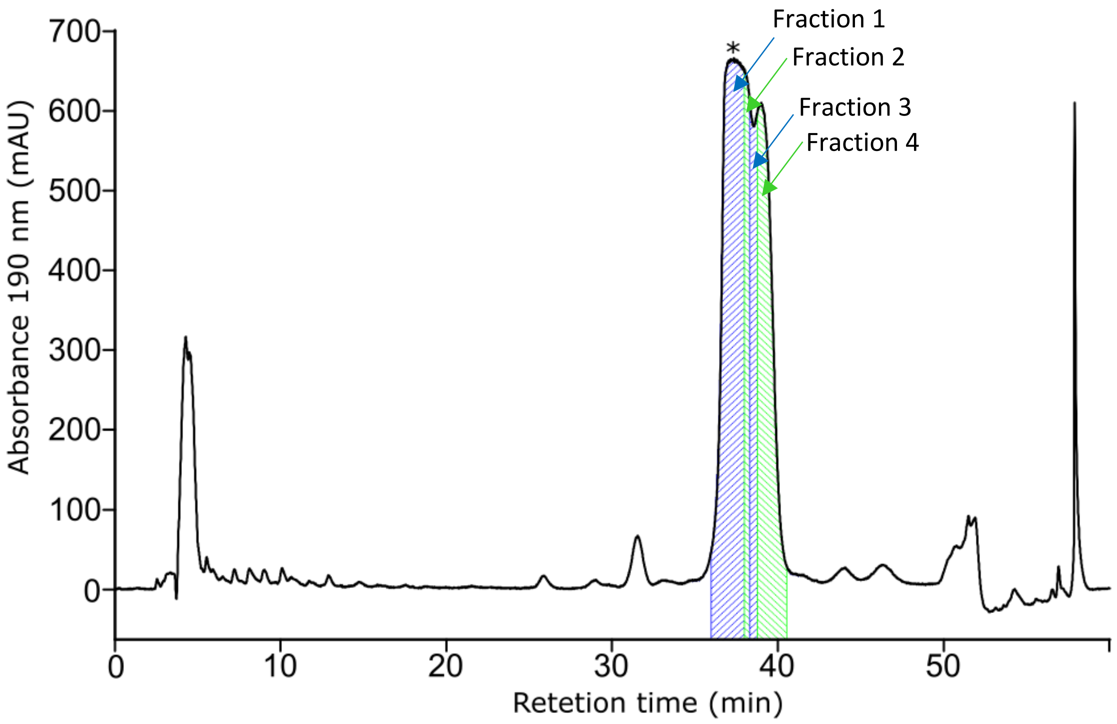

Supplementary Figure 19. Purification of the main cannabinoid from *R. columnifera*. In order to obtain the high degree of purity necessary for NMR structure determination, the cannabinoid containing fraction from the initial preparative HPLC run (Fig. 7C) was further purified by additional preparative HPLC fractionation and biosensor-based cannabinoid determination. Four fractions were collected (green and blue) and fraction 1 (also denoted by a *) was used for NMR.

Supplementary Table 1. List of strains used in this work.

#	Name	Genotype
1	AM254	MAT α, his3, ura3, trp1, lexO:LEU2, rox1, dos2, yer134c, vba5, ygr259c, ynr063w, ubc7

Chassis strains

#	Name	Parent; Mutations
2	KM102	AM254; ΔSTE3-0
3	KM106	AM254; ΔSTE3-0, ΔSST2-0
4	KM108	AM254; ΔSTE3-0, ΔSST2-0, ΔFAR1-0
5	KM109	AM254; ΔSTE3-0, ΔSST2-0, ΔFAR1-0, ΔSTE12-0
6	KM111	AM254; ΔSTE3-0, ΔSST2-0, ΔFAR1-0, ΔSTE12-0, ΔGPA1-0

Biosensor strains

#	Name	Parent	Assembler 1	Assembler 2	Assembler 3	Assembler 4	Assembler 5
7	KM201	KM111;	pX3A EV	pASS2A - P$_{PGK1}$- Gpa1-Gαi1	pASS2B-P$_{RET2}$-STE12	pASS2C -EV	pX3C - P$_{FIG1}$-ZsGREEN
8	KM202	KM111;	pX3A - P$_{CCW12}$-CB$_2$-3XHA	pASS2A - P$_{PGK1}$- Gpa1-Gαi1	pASS2B-P$_{RET2}$-STE12	pASS2C -EV	pX3C - P$_{FIG1}$-ZsGREEN
9	KM203	KM111;	pX3A - P$_{CCW12}$-MFαSS-CB$_2$-3XHA	pASS2A - P$_{PGK1}$- Gpa1-Gαi1	pASS2B-P$_{RET2}$-STE12	pASS2C -EV	pX3C - P$_{FIG1}$-ZsGREEN
10	KM204	KM111;	pX3A - P$_{CCW12}$-MFαSS-CB$_2$-3XHA	pASS2A - P$_{PGK1}$- Gpa1-Gαi1	pASS2B-P$_{RET2}$-STE12	pASS2C - BvCYP76AD1-pSED1/PTDH3-BvDOPA5GT	pX3C - P$_{FIG1}$-DOD
11	KM205	KM111;	pX3A - P$_{CCW12}$-MFαSS-CB$_2$-3XHA	pASS2A - P$_{PGK1}$- Gpa1-Gαi1	pASS2B-P$_{RET2}$-STE12	pASS2C - pHHF2-BvCYP76AD5	pX3C - P$_{FIG1}$-DOD
12	KM206	KM111;	pX3A - P$_{CCW12}$-MFαSS-CB$_2$-3XHA	pASS2A - P$_{PGK1}$- Gpa1-Gαi1	pASS2B-P$_{RET2}$-STE12	pASS2C -EV	pX3C - P$_{FIG1}$-NanoLuc
13	KM207	KM108;	PX3A – P$_{CCW12}$-A$_{2A}$	pB - EV			pX3C - P$_{FIG1}$-NanoLuc

Auxiliary strains

#	Name	Parent	Assembler 1	Assembler 2	Assembler 3	Assembler 4	Assembler 5
14	KM112	KM111;	pX3A - EV	pASS2A - P$_{PGK1}$- Gpa1-Gαi1	pASS2B-P$_{RET2}$-STE12	pASS2C -EV	pX3C - P$_{FIG1}$-NanoLuc
15	KM113	KM111;	pX3A - P$_{CCW12}$-CB$_2$-SfGFP	pASS2A - P$_{PGK1}$- Gpa1-Gαi1	pASS2B-P$_{RET2}$-STE12	pASS2C -EV	pX3C - P$_{FIG1}$-NanoLuc
16	KM114	KM111;	pX3A - P$_{CCW12}$-MFαSS-CB$_2$-SfGFP	pASS2A - P$_{PGK1}$- Gpa1-Gαi1	pASS2B-P$_{RET2}$-STE12	pASS2C -EV	pX3C - P$_{FIG1}$-NanoLuc
17	KM115	KM111;	pX3A - P$_{CCW12}$-CB$_2$-3XHA	pASS2A - P$_{PGK1}$- Gpa1-Gαi1	pASS2B-P$_{RET2}$-STE12	pASS2C -EV	pX3C - P$_{FIG1}$-NanoLuc

Supplementary Table 2. Determination of the Z' score for high-throughput screening assays. The robustness of HTS assays evaluated by calculating the Z' score for CB_2 agonist and antagonist assays. Six replicates of negative and positive control assays were performed with the biosensor strain KM206. For agonist assays, a saturating concentration of a known agonist, CP55940, was used in the positive control and the equivalent amount of DMSO in the negative control. For antagonist assays, a concentration of CP55940 similar to the EC_{50} (2 nM) was used in negative controls. 2 nM CP55940 together with a saturating concentration of the known antagonist CBD (100 µM) was used in the positive control.

	**Output (Luminescence, RLU)**			
	**Agonist assays**		**Antagonist assays**	
**Replicate**	+ve control	-ve control	+ve control	-ve control
**1**	3268319	40521	49698	1008028
**2**	3251599	42599	49445	1291849
**3**	3194136	41276	49786	916747
**4**	3199930	40694	46518	1023581
**5**	2937651	38654	46328	1088882
**6**	3007764	38990	53157	1017596
**µ**	3143233	40456	49155	1057781
**σ**	136975	1464	2519	127204
**Z´**	**0.866**		**0.614**	

Supplementary Table 3. List of screening hits.

Antagonists	Label[a]	SMILES[b]	Manufacturer
AGO1	P1-H4	Cc1c2cc(ccc2oc1C(Nc1ccc(c(c1)OC)NC(c1ccco1)=O)=O)[Cl]	ChemDiv
AGO2	P4-C4	CC(C)C(NC(C([Cl])([Cl])[Cl])NC(Nc1ccccc1C(O)=O)=S)=O	Chembridge
**Agonists**			
ANT1	P1-C10	C1CC2=C(C=C(C#N)C(N2)=O)C(C1)=NNc1ccccc1	Chembridge
ANT2	P3-B11	CCOCc1c(C)cc(C)c(C2C(C#N)=C(N)Oc3c2c(C)n[nH]3)c1C	Chembridge
ANT3	P5-C16	C(CC(O)=O)CNC(c1ccncc1)=O	Chembridge
ANT4	P1-D23	CCc1cc(C(=O)OCC)c(NC(CC2C(Nc3ccccc3N2)=O)=O)s1	ChemDiv
ANT5	P1-F23	CCc1ccc(cc1)NC(CN1C=Nc2c(C1=O)c(cs2)c1ccccc1)=O	ChemDiv
ANT6	P1-N23	Cc1ccc(c(c1)N(=O)=O)N1C(C2C3CCC(C=C3)C2C1=O)=O	ChemDiv
ANT7	P2-I17	CC(c1ccc(cc1)OCCOc1ccc(C#N)cc1)=NO	Chembridge

[a]The label describes the position of each hit in the screening plate.
[b]SMILES is a textual representation of the chemical structure.

Supplementary Table 4. List of plant extracts used for bioprospecting. Label indicates the position of the extract in the screening plate.

#	Label	Species name	Tissue	Source
1	A01	*Agastache mexicana* (Kunth) Lint & Epling (Toronjil morado)	Aerial	[1]
2	A02	*Annona muricata* L. (Guanabana)	Leaves	[1]
3	A03	*Artemisia ludoviciana* Nutt. (Estafiate)	Aerial	[1]
4	A04	*Bougainvillea spectabilis* Wild. (Bugambilia)	Flowers & sepals	[2]
5	A05	*Calendula officinalis* L. (Pot marigold)	Aerial	[3]
6	A06	*Calendula officinalis* L. (Pot marigold)	Flowers	[3]
7	A07	*Calendula officinalis* L. (Pot marigold)	Roots	[3]
8	A08	*Carica papaya* L.	Leaves	[4]
9	A09	*Cascabela thevetia* (L.) Lippold	Leaves	[4]
10	A10	*Casimiroa edulis* La Llave & Lex.	Leaves	[1]
11	A11	*Ceiba pentandra* (L.) Gaertn.	Leaves	[5]
12	A12	*Celosia argentea* var. cristata (L.) Kuntze	Aerial	[6]
13	B01	*Cobaea scandens* Cav.	Leaves	[3]
14	B02	*Cobaea scandens* Cav.	Flower buds	[3]
15	B03	*Coreopsis tinctoria* Nutt.	Aerial	[3]
16	B04	*Coreopsis tinctoria* Nutt.	Roots	[3]
17	B05	*Cosmos bipinnatus* Cav. (Cosmos)	Aerial	[3]
18	B06	*Cosmos bipinnatus* Cav. (Cosmos)	Roots	[3]
19	B07	*Croton incanus* Kunth (Torrey)	Aerial	[1]
20	B08	*Cuphea ignea* A.DC.	Aerial	[3]
21	B09	*Chenopodium graveolens* Wild. (Epazote)	Aerial	[1]
22	B10	*Echinacea purpurea* (L.) Moench	Stem	[6]
23	B11	*Echinacea purpurea* (L.) Moench	Roots	[6]
24	B12	*Echinacea purpurea* (L.) Moench	Leaves	[6]
25	C01	*Equisetum hyemale* L.	Aerial	[1]
26	C02	*Eschscholzia californica* Cham.	Fruits	[3]
27	C03	*Eschscholzia californica* Cham.	Leaves	[3]
28	C04	species of *Euphorbia*	Aerial	[1]
29	C05	*Pseudognaphalium canescens* (DC.) W.A.Weber	Flowers	[1]
30	C06	*Pseudognaphalium canescens* (DC.) W.A.Weber	Stem	[1]
31	C07	*Clarkia amoena* (Lehm.) A.Nelson & J.F.Macbr.	Leaves	[3]
32	C08	*Clarkia amoena* (Lehm.) A.Nelson & J.F.Macbr.	Flowers	[3]
33	C09	*Ipomoea purpurea* L.	Aerial	[6]
34	C10	*Ipomoea purpurea* L.	Roots	[6]
35	C11	*Ipomoea tricolor* Cav.	Leaves	[6]
36	C12	*Ipomoea tricolor* Cav.	Stem	[6]
37	D01	*Ipomoea tricolor* Cav.	Roots	[6]
38	D02	*Amphipterygium adstringens* (Schltdl.) Standl.	Bark	[1]
39	D03	*Justicia spicigera* Schlechtendal	Aerial	[1]

40	D04	*Lippia graveolens* H. B. K.	Aerial	1
41	D05	*Melothria scabra* Naudin	Leaves	6
42	D06	*Mirabilis jalapa* L.	Leaves	3
43	D07	*Mirabilis jalapa* L.	Flowers	3
44	D08	*Nemophila menziesii* Hook. & Arn.	Aerial	6
45	D09	*Persea americana* Mill. (Aguacate)	Leaves	1
46	D10	*Piper auritium* Kunth (Hierba santa)	Leaves	7
47	D11	*Plumeria rubra* L. (Flor de mayo)	Leaves	8
48	D12	*Prosopis laevigata* (Humb. & Bonpl. ex Willd.) M.C.Johnst.	Leaves	9
49	E01	*Pseudobombax ellipticum* (Kunth) Dugand (Coquito/Xiloxochitl)	Leaves	10
50	E02	*Psidium guajava* L. (Guayaba)	Leaves	1
51	E03	*Rhizophora mangle* L. (Mangle)	Stem	1
52	E04	*Rosa ×centifolia* L. (Rosa de castilla)	Flowers	1
53	E05	*Ratibida columnifera* (Nutt.) Wooton & Standl.	Aerial	6
54	E06	*Ratibida columnifera* (Nutt.) Wooton & Standl.	Roots	6
55	E07	*Selaginella lepidophylia* (Hook & Grev.) Spring. (Doradilla)	plant	1
56	E08	*Smilax aristolochiifolia* Mill. (Zarzaparrilla)	Roots	1
57	E09	*Swietenia humilis* Zucc. (Zopilote)	Seeds	1
58	E10	*Tagetes patula* L. (Cempaxochitl)	Leaves	3
59	E11	*Tagetes patula* L. (Cempaxochitl)	Flowers	3
60	E12	*Tecoma stans* (L.) Juss. ex Kunth (Nixtamaxochitl)	Leaves	11
61	F01	*Theobroma cacao* L. (Cacao)	Seeds	12
62	F02	*Tithonia rotundifolia* (Mill.) S.F.Blake (Girasol mexicano)	Stem	6
63	F03	*Tithonia rotundifolia* (Mill.) S.F.Blake (Girasol mexicano)	Roots	6
64	F04	*Tithonia rotundifolia* (Mill.) S.F.Blake (Girasol mexicano)	Leaves	6
65	F05	*Urtica dioica* L. (Ortiga)	Aerial	1
66	F06	*Vachellia farnesiana* (L.) Wight & Arn. (Huizache)	Flowers	9
67	F07	*Zinnia elegans* Jacq. (Cinia)	Flowers	6
68	F08	*Zinnia elegans* Jacq. (Cinia)	Aerial	6
69	F09	*Zinnia elegans* Jacq. (Cinia)	Roots	6
70	F10	species of *Allionia* (Hierba de la hormiga)	Aerial	1
71	F11	species of *Kohleria* (Tlachichinoli)	Leaves	1

[1] Herbolaria El Sauz de Silva

[2] Collected in surroundings of Cd. Lerdo, Durango, Mexico.

[3] Det Biovidenskabelige Fakultets Have, Botanical Garden, University of Copenhagen, Frederiksberg, Denmark

[4] Collected in surroundings of Zona Arquelogica Bocana del Rio Copalita, Oaxaca, Mexico.

[5] Collected in surroundings of Aeropuerto internacional bahias de Huatulco, Oaxaca, Mexico.

[6] Grown from seeds in greenhouse at the Department of Plant and Environmental Sciences, University of Copenhagen Denmark

[7] Collected in surroundings of Crucecita, Oaxaca, Mexico.

[8] Collected in surroundings of San Isidro Roaguia, Hierve el Agua, Oaxaca, Mexico.

[9] Collected in surroundings of Torreon, Coahuila, Mexico.

[10] Collected in surroundings of Oaxaca city, Oaxaca, Mexico.

[11] Collected in surroundings of Playa la entrega, Crucecita, Oaxaca, Mexico.

[12] Raw seeds of Cacao were purchased from distributor TrancePlants in Canada

Supplementary Table 5. Biosensor signal intensity of *R. columnifera* fraction dilutions[a].

		log dilution					
		0	-1	-2	-3	-4	-5
Fraction	1	4832.349	3589.745	3059.277	2608.742	2458.564	1678.606
	2	6280.843	3606.701	2829.165	2264.785	2318.074	1773.073
	3	8894.43	3737.501	2855.81	2281.741	2536.075	2104.918
	4	8506.873	3962.769	2637.809	2245.407	2204.229	2056.473
	5	9422.476	3485.589	2666.875	2204.229	2167.896	2008.029
	6	24856.93	5985.331	2739.542	2356.83	2274.474	2141.251
	7	1576.872	4660.371	2800.098	2390.741	2475.519	2078.273
	8	903.492	11251.26	3846.502	2727.431	2041.94	2201.807
	9	462.646	8538.361	17379.5	2943.01	1664.072	1470.294
	10	7164.957	3880.413	1751.272	1455.76	1567.183	1564.761
	11	4289.77	2153.363	1823.939	1412.16	1320.115	1499.36
	12	3262.744	1918.406	1727.05	1516.316	1455.76	1404.894
	13	2395.586	2027.407	1804.562	1576.872	1588.983	1356.449
	14	3369.322	1995.918	1656.806	1492.094	1605.939	1019.759
	15	5689.819	1988.651	1891.762	1555.072	1448.494	1235.337
	16	3279.7	1610.783	1957.162	1685.872	1753.695	1361.293

[a]The R. columnifera extract was fractionated into 16 fractions and 1-to-10^5-fold dilutions were made of each fraction. The presence of cannabinoids was assayed by incubating each dilution with the strain KM206 (luciferase reporter). Luminescence values are in RLU.

Supplementary Table 6. NMR spectroscopic data (600 MHz, MeOD) of dugesialactone (δ in ppm).

Position	$\delta_{H,}$ mult (J in Hz)	δ_C, type [a]
1	5.51, br. s	125.2, CH
2	2.06, m	25.9, CH_2
3	1.56, m	27.7, CH_2
4	1.67, m	35.8, CH
5	-	37.9, C
6	1.79, dd, J = 5.2, 14.1	39.5, CH_2
	1.63, m	
7	3.02, m	37.5, CH
8	4.56, m	80.5, CH
9	2.56, dd, J = 7.8, 12.1	35.3, CH_2
	2.30	
10	-	139.5, C
11	-	141.0, C
12	-	172.4, C
13	6.16, d, J = 2.3	122.3, CH_2
	5.70, d, J = 2.0	
14	0.94, s	20.7, CH_3
15	0.96, d, J = 6.8	16.0, CH_3

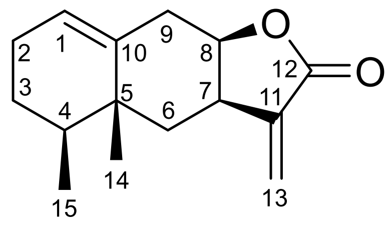

[a] ^{13}C NMR shifts were obtained from HSQC and HMBC spectra.

Supplementary Table 7. The biosensor retains most of its activity after one month of storage. Maximal biosensor activity of the biosensor strain KM206 preserved with 0.1 M K-phosphate pH 7, 0.1% ascorbic acid and nitrogen bubbling was measured by inducing with 100 µM THC before and after 31 days of storage at +4°C.

	**Preserved KM206**	
	**0 d**	**31 d**
Replicate	Luminescence (RLU)	
1	1650490	1396851
2	1481308	1340614
3	1395698	1405596
µ	1509165	1381020
σ	129660	35265
**Activity %**	100.00	91.51

Supplementary Table 8. Plasmid construction. All plasmids were construction by USER cloning combining one single-promoter and one ORF (made from one or two fragments) or a two-sided promoter and two ORFs. PCR fragments used here are described in Supplementary Table 9.

plasmid name	Backbone	ORF 1	promoter	ORF 2
pX3A -EV	pX3A			
pX3A - P_{CCW12}-CB_2-3XHA	pX3A	CB_2-3XHA	P_{CCW12}	
pX3A - P_{CCW12}-MFαSS-CB_2-3XHA	pX3A	MFαSS-CB_2-3XHA	P_{CCW12}	
pX3A - P_{CCW12}-CB_2-SfGFP	pX3A	CB_2-linker + SfGFP	P_{CCW12}	
pX3A - P_{CCW12}-MFαSS-CB_2-SfGFP	pX3A	MFαSS-CB_2-linker + SfGFP	P_{CCW12}	
pASS2A- EV	pASS2A			
pASS2A - P_{PGK1}- Gpa1-Gαi1	pASS2A	GPA1-GAi1	P_{PGK1}	
pASS2B-EV	pASS2B			
pASS2B- P_{RET2}-STE12	pASS2B	STE12	P_{RET2}	
pASS2C -EV	pASS2C			
pASS2C - P_{HHF2}-BvCYP76AD5	pASS2C	*Bv*CYP76AD5	P_{HHF2}	
pASS2C - BvCYP76AD1- P_{SED1}/P_{TDH3}-BvDOPA5GT	pASS2C	*Bv*CYP76AD1	P_{SED1}/P_{TDH3}	*Bv*DOPAGT
pX3C- EV	pX3C			
pX3C - P_{FIG1}-ZsGREEN	pX3C	*Zs*GREEN	P_{FIG1}	
pX3C - P_{FIG1}-DOD	pX3C	*Mj*DOD	P_{FIG1}	
pX3C - P_{FIG1}-NanoLuc	pX3C	NanoLuc	P_{FIG1}	
pB-EV	pB			

Supplementary Table 9. USER fragments used for plasmid construction. These fragments were amplified using primers listed in Supplementary Table 10.

Fragment	Primer-F	Primer-R	Template
CB_2-3XHA	CB_2-F	3XHA-R	pYX222-P_{TPI}-CNR2
MFαSS-CB_2-3XHA	MFα-F	CB_2-R	pYX222-P_{TPI}-CNR2
CB_2-linker	CB_2-F	CB_2-linker-R	pYX222-P_{TPI}-CNR2
MFαSS-CB_2-linker	MFα-F	CB_2-linker-R	pYX222-P_{TPI}-CNR2
SfGFP	sfGFP-linker-F	sfGFP-R	iGEM distribution kit 2019
GPA1-Gαi1	GPA1-F	GPA1/Gα1-R	yeast genomic DNA
STE2	STE12-F	STE12-R	yeast genomic DNA
BvCYP76AD5	CYP76AD5-F	CYP76AD5-R	Synthetic DNA
BvCYP76AD1	CYP76AD1-F	CYP76AD1-R	Synthetic DNA
ZsGREEN	ZsGREEN-F	ZsGREEN-R	Synthetic DNA
MjDOD	USER-DOD-F	USER-DOD-R	Synthetic DNA
BvDOPA5GT	DOPA5GT-F	DOPA5GT-R	Synthetic DNA
NanoLuc	NanoLuc-F	NanoLuc-R	Synthetic DNA
P_{CCW12}	PCCW12-F	PCCW12-R	yeast genomic DNA
P_{PGK1}	PPGK1-F	PPGK1-R	yeast genomic DNA
P_{RET2}	PRET2-F	PRET2-R	yeast genomic DNA
P_{HHF2}	PHHF2-F	PHHF2-R	yeast genomic DNA
P_{SED1}/P_{TDH3}	PTDH3-F	PSED1-R	yeast genomic DNA
P_{FIG1}	PFIG1-F	PFIG1-R	yeast genomic DNA
STE3 KO	STE3-OL5	STE3-OL3	pUG72
SST2 KO	SST3-OL5	SST3-OL3	pUG72
FAR1 KO	FAR1-OL5	FAR1-OL3	pUG72
STE12 KO	STE12-OL5	STE12-OL3	pUG72
GPA1 KO	GPA1-OL5	GPA1-OL3	pUG72

Supplementary Table 10. PCR primers used in this work

1	CB_2-F	ATCAACGGGUAAAATGGAGGAATGCTGGGTGA
2	3XHA-R	ATCAACGGGUAAAATGAGATTTCCTTCAATTTTTACTGCAGTT
3	MFα-F	ATCAACGGGUAAAATGAGATTTCCTTCAATTTTTACTGCAGTT
4	GPA1 F	ATCAACGGGUAAAATGGGGTGTACAGTGAGTACGCAAA
5	GPA1/Gα1 R	CGTGCGAUTCAAAACAAACCACAATCTTTAAGGTTTTGCTGGATGATTAGA
6	STE12 F	ATCAACGGGUAAAATGAAAGTCCAAATAACCAATAGTAGAAC
7	STE12 R	CGTGCGAUTCAGGTTGCATCTGGAAGGTTTTTAT
8	CYP76AD1F	ATCAACGGGUAAAATGGATCATGCTACTTTGGCTATGA
9	CYP76AD1R	CGTGCGAUCTATTAGTATCTTGGAATGGGGATCAACT
10	CYP76AD5F	ATCAACGGGUAAAATGGATAACACTACCTTGGCTTTGA
11	CYP76AD5R	CGTGCGAUCTATTATTTTCTTGGAACGGGAATAACTTG
12	DOPA5GT-F	AGCGATACGUAAAATGACTGCTATTAAGATGAACACT
13	DOPA5GT-R	CACGCGAUTTATTGTAAAGATGGTTCCAACTTGGCT
14	USER-DOD-F	ATCAACGGGUAAAATGAAGGGTACTTACTACATTAACCA
15	USER-DOD-R	CGTGCGAUTTAATCAGTCTTTTGAGTAGTGGGA
16	NanoLuc-F	ATCAACGGGUAAAATGGTTTTTACTTTGGAAGATTTCG
17	NanoLuc-R	CGTGCGAUTACAGGATCTCTTCGAATAATCTATAACCAGTAACGGAGTTAATGGTGACTC
18	ZsGREEN-F	ATCAACGGGUAAAATGGCTCAATCTAAGCATGGTTTGA
19	ZsGREEN-R	CGTGCGAUTTATGGCAAAGCAGAACCAGAAGC
20	PCCW12-F	CACGCGAUTGAACCACACGGTTAGTCCAAAAGG
21	PCCW12-R	ACCCGTTGAUTATTGATATAGTGTTTAAGCGAATGACAGAAG
22	PPGK1-F	CACGCGAUGTGAGTAAGGAAAGAGTGAGGAACT
23	PPGK1-R	ACCCGTTGAUTGTTTTATATTTGTTGTAAAAAGT
24	PRET2-F	CACGCGAUACGATGGCTTCTTATCTCACTTCAA
25	PRET2-R	ACCCGTTGAUTGTGTATTTCTTTTGATGGAGCTATAG
26	PHHF2-F	CACGCGAUTGTGGAGTGTTTGCTTGGATTCTTTAG
27	PHHF2-R	ACCCGTTGAUTATTTTATTGTATTGATTGTTGTTTTTGCTACTCT
28	PFIG1-F	CACGCGAUGAACTGGTTGATATTATTACTGGTG
29	PFIG1-R	ACCCGTTGAUTTTTTTTTTTTTTTTTTTTGTTTGTTTGTTTGTTTGTTTAC
30	PTDH3-F	CACGCGAUCAGTTCGAGTTTATCATTATCAATACTGCC
31	PSED1-R	ACCCGTTGAUCTTAATAGAGCGAACGTATTTTATTTTGCTTG
32	STE3-OL5	TAGAGGCAATTAAATTTGTGTAGGAAAGGCAAAATACTATCAAAATTTTCCAGCTGAAGCTTCGTACGC
33	STE3-OL3	GTAAAAATAAAATACTCCTAGTCCAGTAAATATAATGCGACACTCTTGTGGCATAGGCCACTAGTGGATCTG

34	FAR1-OL5	GTCTATAGATCCACTGGAAAGCTTCGTGGGCGTAAGAAGGCAATCTATTACAGCTGAAGCTTCGTACGC
35	FAR1-OL3	TTACGACGCATTATATATATTCAGTCATTGCGTAGTATAGACGTGGAGAAGCATAGGCCACTAGTGGATCTG
36	SST2-OL5	TATCTGAGGCGTTATAGGTTCAATTTGGTAATTAAAGATAGAGTTGTAAGCAGCTGAAGCTTCGTACGC
37	SST2-OL3	AGGACTGTTTGTGCAATTGTACCTGAAGATGAGTAAGACTCTCAATGAAAGCATAGGCCACTAGTGGATCTG
38	STE12-OL5	ATAATGAAAACGATGAAGTCAGTAAAGCTACTCCGGGCGAAGTTGAAGAACAGCTGAAGCTTCGTACGC
39	STE12-OL3	GAATGAGCTCCACCTTCTTCTGACTGGACCACCAAAGTATTGGAATCCGGGCATAGGCCACTAGTGGATCTG
40	GPA1-OL5	AGAACAAAAGAGCCAATGATGTCATCGAGCAATCGTTGCAGCTGGAGAAACAGCTGAAGCTTCGTACGC
41	GPA1-OL3	CTCAATACGAACTTCATAGTTTGGGTATCGGTAGCGCAGGTTCGTTTCACGCATAGGCCACTAGTGGATCTG
42	sfGFP-linker-F	AGGTGGCUCTGGTGGTGGATCAATGCGTAAAGGCGAAGAGCT
43	sfGFP-R	CGTGCGAUTCATTTGTACAGTTCATCCATACCAT
44	CB_2-linker-R	AGCCACCUCCCTGAGCAGCGTAATCTGGAA
45	B-M-R:	CTAATAGCCACCTGCATTGG
46	STE3-A-F	CTGGGTATGGGTGCTAATTT
47	STE3-B-R	CAATAAGTTTTGGCAACCGT
48	FAR1-A F:	TTGTTAGGCGGGCAAGAGAGACC
49	FAR1-B-R	TCCAGGCTGATCATGAAAGTTGGTG
50	SST2-A-F	CGTTAGAATTGTTGTCTGTC
51	SST2-B-R	AGAACTTTCTCTTTTGGCTC
52	STE12-A-F	TAAGCACTGAAGACTTGTTT
53	STE12-B-R	CACAAGAGACAAAGCCTTGT
54	GPA1-F	TGTCACTCCGTTTCTAACA
55	GPA1-R	CTGTAACCTTTCTTGATGGGA

Supplementary Table 11. Sequences of synthetic DNA fragments used in this work.

MfαSS-CNR2-3XHA	ATGAGATTTCCTTCAATTTTTACTGCAGTTTTATTCGCAGCATCCTCCGCATTA<br>GCTGCTCCAGTCAACACTACAACAGAAGATGAAACGGCACAAATTCCGGCTG<br>AAGCTGTCATCGGTTACTCAGATTTAGAAGGGGATTTCGATGTTGCTGTTTTGC<br>CATTTTCCAACAGCACAAATAACGGGTTATTGTTTATAAATACTACTATTGCCA<br>GCATTGCTGCTAAAGAAGAAGGGGTATCTCTCGAAAAAAGAGAGGCTGGATC<br>CATGGAGGAATGCTGGGTGACAGAGATAGCCAATGGCTCCAAGGATGGCTTG<br>GATTCCAACCCTATGAAGGATTACATGATCCTGAGTGGTCCCCAGAAGACAGC<br>TGTTGCTGTGTTGTGCACTCTTCTGGGCCTGCTAAGTGCCCTGGAGAACGTGG<br>CTGTGCTCTATCTGATCCTGTCCTCCCACCAACTCCGCCGGAAGCCCTCATACC<br>TGTTCATTGGCAGCTTGGCTGGGGCTGACTTCCTGGCCAGTGTGGTCTTTGCAT<br>GCAGCTTTGTGAATTTCCATGTTTTCCATGGTGTGGATTCCAAGGCTGTCTTCC<br>TGCTGAAGATTGGCAGCGTGACTATGACCTTCACAGCCTCTGTGGGTAGCCTC<br>CTGCTGACCGCCATTGACCGATACCTCTGCCTGCGCTATCCACCTTCCTACAAA<br>GCTCTGCTCACCCGTGGAAGGGCACTGGTGACCCTGGGCATCATGTGGGTCCT<br>CTCAGCACTAGTCTCCTACCTGCCCCTCATGGGATGGACTTGCTGTCCCAGGC<br>CCTGCTCTGAGCTTTTCCCACTGATCCCCAATGACTACCTGCTGAGCTGGCTCC<br>TGTTCATCGCCTTCCTCTTTTCCGGAATCATCTACACCTATGGGCATGTTCTCT<br>GGAAGGCCCATCAGCATGTGGCCAGCTTGTCTGGCCACCAGGACAGGCAGGT<br>GCCAGGAATGGCCCGAATGAGGCTGGATGTGAGGTTGGCCAAGACCCTAGGG<br>CTAGTGTTGGCTGTGCTCCTCATCTGTTGGTTCCCAGTGCTGGCCCTCATGGCC<br>CACAGCCTGGCCACTACGCTCAGTGACCAGGTCAAGAAGGCCTTTGCTTTCTG<br>CTCCATGCTGTGCCTCATCAACTCCATGGTCAACCCTGTCATCTATGCTCTACG<br>GAGTGGAGAGATCCGCTCCTCTGCCCATCACTGCCTGGCTCACTGGAAGAAGT<br>GTGTGAGGGGCCTTGGGTCAGAGGCAAAAGAAGAAGCCCCGAGATCCTCAGT<br>CACCGAGACAGAGGCTGATGGGAAAATCACTCCGTGGCCAGATTCCAGAGAT<br>CTAGACCTCTCTGATTGCACTAGGATCTTTTACCCATACGATGTTCCTGACTAT<br>GCGGGCTATCCCTATGACGTCCCGGACTATGCAGGTTCCTATCCATATGACGT<br>TCCAGATTACGCTGCTCAGTGA
NanoLuc	ATGGTTTTTACTTTGGAAGATTTCGTTGGTGATTGGGAACAAACTGCTGCTTAC<br>AATTTGGATCAAGTCTTGGAACAAGGTGGTGTCTCTTCTTTATTGCAAAACTTG<br>GCTGTTTCTGTCACCCCAATTCAAAGAATAGTTAGGTCTGGTGAAAACGCCTT<br>GAAGATCGATATTCATGTTATCATCCCATACGAAGGTTTGTCCGCTGATCAAA<br>TGGCTCAAATTGAAGAAGTTTTCAAGGTTGTTTACCCAGTTGATGATCACCAC<br>TTCAAGGTTATTTTGCCATACGGTACTTTGGTTATTGATGGTGTTACTCCCAAC<br>ATGCTGAATTACTTTGGTAGACCTTATGAAGGTATCGCCGTTTTTGATGGTAAG<br>AAGATTACTGTTACTGGCACTTTGTGGAACGGTAACAAGATTATTGACGAAAG<br>GTTGATCACTCCAGACGGTTCTATGTTATTCAGAGTCACCATTAACTCCGTTAC<br>TGGTTATAGATTATTCGAAGAGATCCTGTAA
ZsGreen	ATGGCTCAATCTAAGCATGGTTTGACTAAGGAAATGACTATGAAGTACAGAAT<br>GGAAGGTTGTGTTGACGGTCATAAGTTCGTTATCACTGGTGAAGGAATTGGTT<br>ATCCATTCAAAGGTAAGCAGGCTATCAACTTGTGTGTTGTTGAAGGTGGTCCA<br>TTGCCATTCGCTGAAGACATCTTGTCTGCTGCTTTCATGTACGGTAACAGAGTC<br>TTCACTGAATACCCACAAGACATTGCTGATTACTTCAAGAACTCTTGTCCAGC<br>TGGTTACACTTGGGATAGATCCTTCTTGTTTGAAGATGGTGCTGTTTGTATCTG<br>TAATGCTGATATCACTGTTTCTGTTGAAGAAAACTGTATGTATCATGAATCTAA<br>GTTCTATGGTGTCAACTTCCCAGCTGATGGTCCAGTTATGAAGAAGATGACTG<br>ATAACTGGGAACCATCTTGTGAAAAGATCATTCCAGTTCCAAAGCAAGGTATC<br>TTGAAAGGTGATGTTTCTATGTACTTGCTATTGAAAGATGGTGGTAGATTGAG<br>ATGTCAATTCGATACTGTCTACAAAGCTAAGTCTGTTCCAAGAAAGATGCCAG<br>ATTGGCATTTCATTCAACATAAGTTGACTAGAGAAGATAGATCTGATGCTAAG

	AATCAAAAGTGGCATCTAACTGAACATGCTATTGCTTCTGGTTCTGCTTTGCCA<br>TAA
SfGFP	ATGCGTAAAGGCGAAGAGCTGTTCACTGGTGTCGTCCCTATTCTGGTGGAACT<br>GGATGGTGATGTCAACGGTCATAAGTTTTCCGTGCGTGGCGAGGGTGAAGGTG<br>ACGCAACTAATGGTAAACTGACGCTGAAGTTCATCTGTACTACTGGTAAACTG<br>CCGGTACCTTGGCCGACTCTGGTAACGACGCTGACTTATGGTGTTCAGTGCTTT<br>GCTCGTTATCCGGACCATATGAAGCAGCATGACTTCTTCAAGTCCGCCATGCC<br>GGAAGGCTATGTGCAGGAACGCACGATTTCCTTTAAGGATGACGGCACGTAC<br>AAAACGCGTGCGGAAGTGAAATTTGAAGGCGATACCCTGGTAAACCGCATTG<br>AGCTGAAAGGCATTGACTTTAAAGAAGACGGCAATATCCTGGGCCATAAGCT<br>GGAATACAATTTTAACAGCCACAATGTTTACATCACCGCCGATAAACAAAAA<br>AATGGCATTAAAGCGAATTTTAAAATTCGCCACAACGTGGAGGATGGCAGCG<br>TGCAGCTGGCTGATCACTACCAGCAAAACACTCCAATCGGTGATGGTCCTGTT<br>CTGCTGCCAGACAATCACTATCTGAGCACGCAAAGCGTTCTGTCTAAAGATCC<br>GAACGAGAAACGCGATCATATGGTTCTGCTGGAGTTCGTAACCGCAGCGGGC<br>ATCACGCATGGTATGGATGAACTGTACAAATGA
BvCYP76AD1	ATGGATCATGCTACTTTGGCTATGATTTTGGCCATCTTGTTCATCTCATTCCAC<br>TTCATCAAGCTGTTGTTCTCTCAACAAACCACTAAGTTGTTGCCACCAGGTCCT<br>AAACCATTGCCAATTATTGGTAACATCTTGGAGGTTGGTAAAAAGCCACATAG<br>ATCCTTTGCTAACTTGGCTAAAATTCACGGTCCATTGATCTCATTGAGATTGGG<br>TTCTGTTACTACCATCGTTGTTTCTTCAGCTGATGTTGCCAAAGAGATGTTCTT<br>GAAAAAGGATCACCCATTGTCCAACAGAACCATTCCAAATTCTGTTACAGCTG<br>GTGATCATCACAAGTTGACTATGTCTTGGTTGCCAGTTTCTCCAAAGTGGCGTA<br>ATTTCAGAAAGATTACCGCTGTTCATTTGTTGTCCCCACAAAGATTGGATGCTT<br>GTCAAACTTTTAGACACGCTAAGGTTCAACAGTTGTACGAATACGTTCAAGAA<br>TGTGCTCAAAAAGGTCAAGCCGTTGATATTGGTAAAGCTGCTTTTACTACCTC<br>CTTGAACTTGCTGTCCAAGTTGTTTTTCTCTGTTGAATTGGCCCATCATAAGTC<br>CCATACTTCTCAAGAGTTCAAAGAGTTGATCTGGAACATCATGGAAGATATCG<br>GTAAGCCAAATTACGCTGATTACTTCCCAATTTTGGGTTGCGTTGATCCATCTG<br>GTATTAGAAGAAGATTGGCTTGCTCTTTCGATAAGTTGATTGCTGTTTTCCAAG<br>GTATCATCTGTGAAAGATTAGCCCCAGATTCTTCTACTACTACAACTACTACC<br>ACTGATGATGTTTTGGATGTGTTGTTGCAGTTGTTCAAGCAAAACGAATTGAC<br>CATGGGTGAAATCAACCACTTGTTGGTTGATATTTTCGATGCTGGTACTGATAC<br>CACTTCTTCTACATTTGAATGGGTTATGACCGAGTTGATCAGAAACCCAGAAA<br>TGATGGAAAAAGCCCAAGAAGAAATCAAGCAGGTTTTGGGTAAAGACAAGCA<br>GATCCAAGAATCCGATATTATCAACTTGCCATACTTGCAGGCCATCATCAAAG<br>AAACTTTGAGATTGCATCCACCAACCGTTTTTTTGTTGCCAAGAAAAGCTGAT<br>ACCGATGTTGAGTTGTATGGTTACATCGTTCCAAAGGATGCCCAAATCTTGGT<br>TAATTTGTGGGCTATTGGTAGAGATCCAAATGCTTGGCAAAACGCCGATATTT<br>TCTCACCAGAAAGGTTCATTGGTTGCGAAATTGATGTTAAGGGTAGAGACTTT<br>GGCTTGTTGCCATTTGGTGCTGGTAGAAGGATTTGTCCAGGTATGAATTTGGCT<br>ATCAGAATGTTGACTTTGATGCTGGCTACTCTGTTGCAATTTTTCAACTGGAAA<br>TTGGAGGGTGACATCTCACCAAAAGATTTGGATATGGACGAAAAGTTCGGTAT<br>CGCCTTGCAAAAAACTAAGCCATTGAAGTTGATCCCCATTCCAAGATACGGTT<br>CTTAG
BvCYP76AD5	ATGGATAACACTACCTTGGCTTTGATTCTGTCCTCTTTGTTCGTTTGCTTCCAAT<br>TGATCAGGTCCTTCATTAACCATGCCAAGAAGTCTAACAAATTGCCACCAGGT<br>CCTAAGAGAATGCCAATTTTTGGTAACATCTTCGACTTGGGTGAAAAGCCACA<br>TAGATCCTTTGCTAACTTGGCTAAAATTCACGGTCCATTGGTTTCATTGCAATT<br>GGGTTCTGTTACTACCGTTGTTGTTTCTTCTGCTGATGTTGCCAAAGAAATGTT<br>CTTGAAGAACGATCAAGCTTTGGCCAACAGAACTATTCCAGATTCTGTTAGAG<br>CTGGTGATCACGATAAGTTGTCTATGTCTTGGTTGCCAGTTTCTGCTAAATGGC

	GTAACTTGAGAAAGATTTCTGCTGTCCAACTGTTGTCTACCCAAAGATTGGAT GCTTCTCAAGCTCATAGACAATCTAAGGTTCAACAGTTGTTGGAATACGTTCA CGATTGCTCTAAAAAGGGTCAACCAGTTGATATTGGTAGAGCTGCTTTTACTA CCTCCTTGAACTTGTTGTCTAACACCTTCTTCTCTGTTGAATTGGCCTCTCATG AATCTTCAGCTTCCCAAGAGTTTAAGCAATTGATGTGGAACATCATGGAAGAA ATCGGTAGACCAAATTACGCCGATTTTTTCCCAATCTTGGGTTACTTAGATCCA TTCGGTATCAGAAGAAGATTGGCTGGTTATTTCGACCAATTGATTGCCGTTTTC CAAGACATTATTGGTGAGAGACAAAAGATCAGATCCGCTAATTTGTCTGGTGG TAAGCAAACTACCAACGATATTTTGGATACCCTGCTGAACTTGTACGACGAGA AAGAATTATCTATGGGTGAGATCAACCACCTGTTGGTTGATATTTTTGATGCTG GTACTGATACCACTGCTTCTACTTTGGAATGGGCTATGGCTGAATTGGTTAAG AATCCAGATATGATGGTTAAGGTCCAGGACGAAATTGAACAAGCTATTGGTA AAGGTTGCTCCATGGTTCAAGAATCTGATATCTCTAAGTTGCCATACTTGCAG GCCATTATCAAAGAAACCTTGAGATTGCATCCACCAACCGTTTTTTTGTTGCCT AGAAAAGCTGATGCAGATGTTGAGTTGTATGGTTACGTTGTTCCAAAGAATGC TCAAGTCTTGGTTAACTTGTGGGCTATAGGTAGAGATCCAAAGGTTTGGAAAA ACCCAGAAGTTTTCTCACCAGAAAGGTTCTTGGAATCCAACATTGATTACAAG GGTAGAGACTTTGAGTTGTTGCCATTTGGTGCTGGTAGAAGAATTTGTCCAGG TTTGACTTTGGCTTACAGGATGTTGAATTTGATGATGGCCAACTTCTTGCACTC TTACGATTGGAAATTGGAAGATGGTATGCACCCTAAGGATTTGGATATGGACG AAAAGTTTGGTATCACCTTGCAAAAGGTTAAGCCATTGCAAGTTATTCCCGTT CCAAGAAAATAG
MjDOD	ATGAAGGGTACTTACTACATTAACCATGGTGACCCATTGATGTACTTGAAGAA GCACATTAAGTTGAGGCAGTTTTTGGAAGGTTGGCAAGAAAACGTTGTTATCG AAAAGCCAAAGTCCATCTTGATTATTTCTGCTCATTGGGATACCAACGTTCCA ACTGTTAATTTCGTTGAACATTGCGATACCATCCACGATTTTGATGATTACCCA GATCCACTGTACCAAATTCAGTATAGAGCCCCAGGTGCTCCTAATTTGGCTAA AAAAGTTGAAGAGTTGCTGAAAGAATCCGGTATGGAATGTGAAATCGATACC AAAAGAGGTTTGGATCACGCTGCTTGGTTTCCACTAATGTTTATGTATCCAGA AGCCAACATTCCAATCTGCGAATTGTCTGTTCAACCATCCAAAGATGGTATCC ATCATTACAATGTTGGTAAGGCTTTGTCCCCATTATTGCAACAAGGTGTTTTGA TTATTGGCTCCGGTGGTACTGTTCATCCATCTGATGATACTCCACATTGTCCAA ATGGTGTTGCTCCATGGGCTATTGAATTTGATAATTGGTTGGAAGATGCCTTGT TGTCCGGTAGATATGAAGATGTCAACAACTTCAAAAAGTTGGCCCCAAACTGG GAAATTTCTCATCCAGGTCAAGAACACTTGTACCCATTGCATGTTGCTTTGGGT GCTGCTGGTAAAAATCCAAAGACTCAATTGATCCATAGATCCTGGGCTGCTAA TGGTGTTTTTGGTTACTCTACTTACAACTTCACTCCCACTACTCAAAAGACTGA TGGTTCATAA
BvDOPA5GT	ATGACTGCTATTAAGATGAACACTAATGGTGAAGGTGAAACCCAACACATTTT GATGATTCCATTCATGGCTCAAGGTCATTTGAGGCCATTTTTGGAATTGGCTAT GTTCCTGTACAAGAGATCCCATGTTATTATCACCTTGTTGACTACTCCATTGAA CGCTGGTTTTTTGAGACATTTGTTGCATCACCACTCCTACTCTTCATCTGGTATT AGAATAGTCGAGCTGCCATTCAATTCTACCAATCATGGTTTGCCACCAGGTAT TGAAAACACTGATAAGTTGACTTTGCCCCTGGTTGTTTCTTTGTTCCATTCCAC CATTTCTTTGGACCCACATTTGAGAGATTACATCTCCAGACATTTTTCACCAGC TAGACCACCATTGTGCGTTATTCATGATGTTTTCTTAGGTTGGGTTGATCAAGT TGCTAAGGATGTTGGTTCTACTGGTGTTGTTTTTACTACTGGTGGTGCTTATGG TACTTCTGCCTATGTTTCTATTTGGAACGATTTGCCACACCAGAACTACTCTGA TGATCAAGAATTTCCATTGCCAGGTTTTCCAGAAAACCATAAGTTCAGAAGAT CCCAGTTGCACAGATTTTTGAGATATGCTGATGGTTCCGATGACTGGTCTAAA TACTTTCAACCACAATTGAGGCAGTCCATGAAGTCTTTTGGTTGGTTGTGTAAC TCCGTGGAAGAAATTGAAACTTTGGGCTTCTCCATCTTGAGAAACTACACTAA GTTGCCAATTTGGGGTATTGGTCCATTGATTGCTTCTCCAGTTCAACATTCTTC

	CTCCGATAACAATTCTACAGGTGCCGAATTTGTTCAGTGGTTGTCTTTGAAAG AACCAGACTCTGTCTTGTACATCTCATTCGGTTCTCAAAACACTATCTCCCCAA CTCAAATGATGGAATTAGCTGCTGGTTTGGAATCCTCTGAAAAACCATTTTTGT GGGTTATTAGAGCCCCATTCGGTTTCGATATTAACGAAGAAATGAGGCCAGAA TGGTTGCCAGAAGGTTTTGAAGAAAGAATGAAGGTCAAGAAGCAGGGCAAAT TGGTTTACAAATTAGGTCCACAGCTGGAAATCTTGAACCACGAATCTATTGGT GGTTTCTTGACTCATTGCGGTTGGAACTCTATTTTGGAGTCTTTGAGAGAAGGT GTCCCAATGTTAGGTTGGCCATTGGCTGCTGAACAAGCTTACAATTTGAAATA CTTGGAGGACGAAATGGGTGTTGCTGTTGAATTAGCTAGAGGTTTGGAGGGTG AAATCTCTAAAGAAAAGGTTAAGAGGATCGTCGAGATGATCTTGGAAAGAAA CGAAGGTTCTAAAGGTTGGGAAATGAAGAACAGAGCTGTTGAGATGGGTAAA AAGTTGAAGGATGCCGTTAACGAGGAAAAAGAATTGAAAGGTTCTTCCGTTA AGGCCATCGATGATTTTTTGGATGCTGTTATGCAAGCCAAGTTGGAACCATCT TTACAATAA

Uncropped image for Supplementary Figure 5: Scan of the westernblot membrane

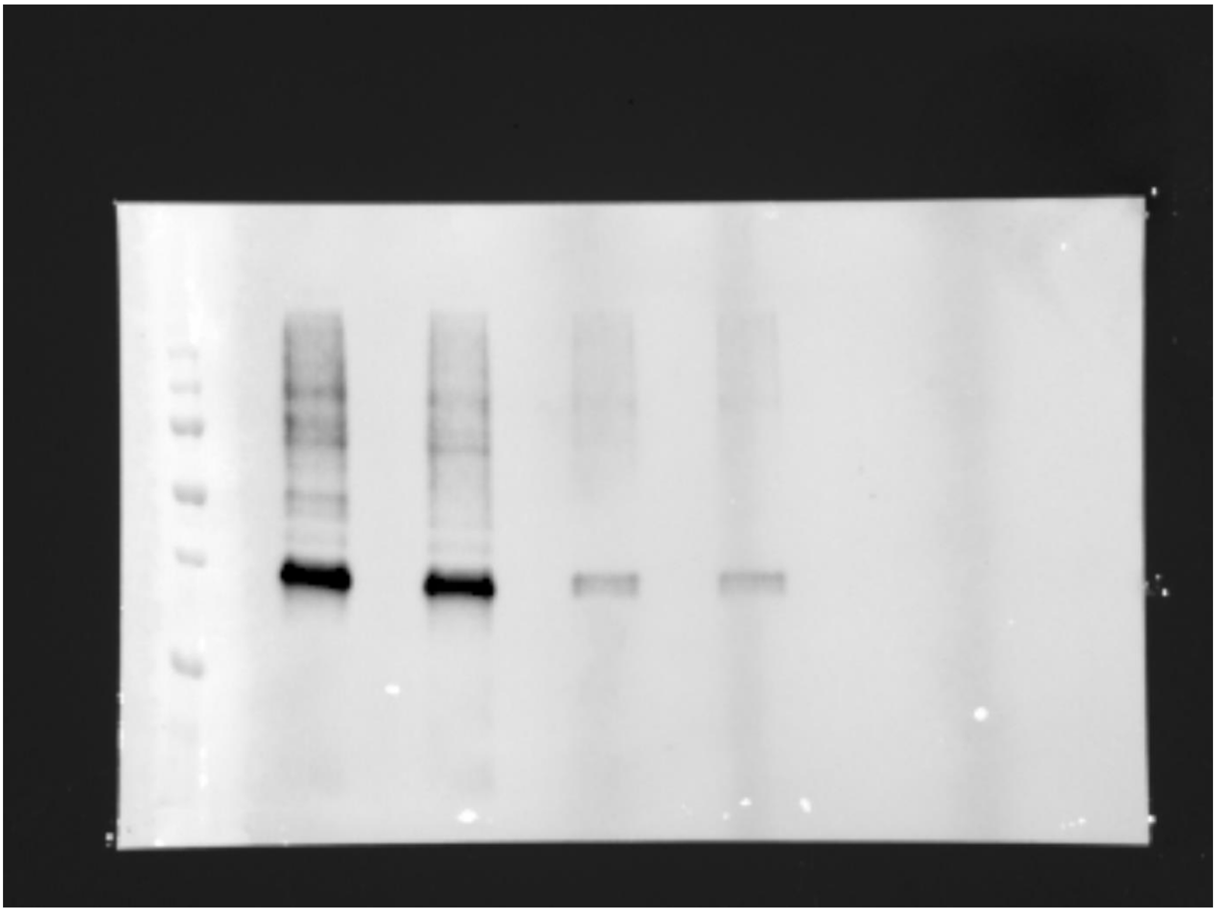

Uncropped image for Supplementary Figure 5: Scan of the SDS-PAGE gel.

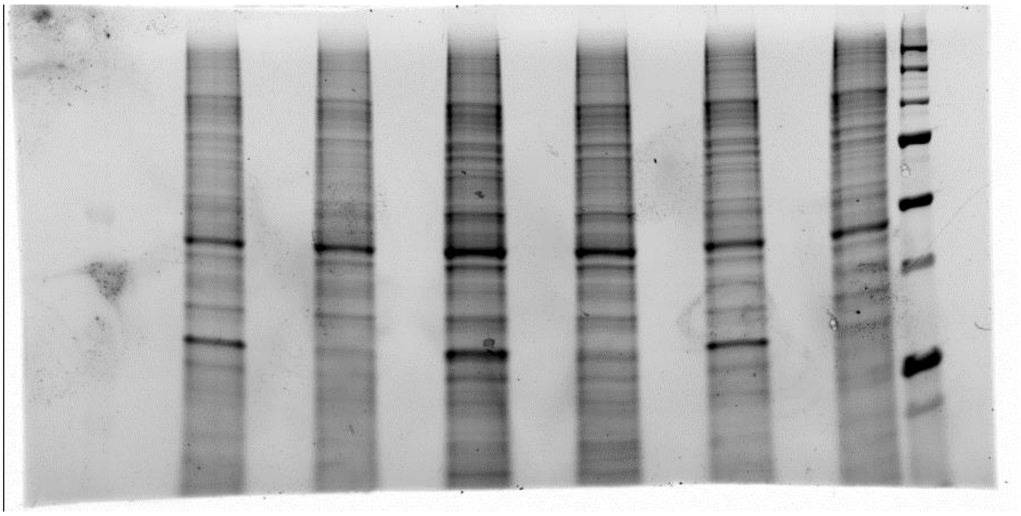

Supplement: Supplementary file 1 — Supplementary information [file 41467_2022_31357_MOESM1_ESM.pdf]
