# Supplementary material for: A GPCR-based yeast biosensor for biomedical, biotechnological, and point-of-use cannabinoid determination
Source: Nat Commun. 2022 Jun 27;13:3664. doi: 10.1038/s41467-022-31357-6 (PMC9237071; doi:10.1038/s41467-022-31357-6)
Supplement: Supplementary file 2 — Reporting Summary [file 41467_2022_31357_MOESM2_ESM.pdf]

nature portfolio

Corresponding author(s): Sotirios C. Kampranis

Last updated by author(s): May 29, 2022

Reporting Summary

Nature Portfolio wishes to improve the reproducibility of the work that we publish. This form provides structure for consistency and transparency in reporting. For further information on Nature Portfolio policies, see our Editorial Policies and the Editorial Policy Checklist.

Statistics

For all statistical analyses, confirm that the following items are present in the figure legend, table legend, main text, or Methods section.

n/a	Confirmed	
☐	☒	The exact sample size (*n*) for each experimental group/condition, given as a discrete number and unit of measurement
☐	☒	A statement on whether measurements were taken from distinct samples or whether the same sample was measured repeatedly
☐	☒	The statistical test(s) used AND whether they are one- or two-sided<br>*Only common tests should be described solely by name; describe more complex techniques in the Methods section.*
☒	☐	A description of all covariates tested
☒	☐	A description of any assumptions or corrections, such as tests of normality and adjustment for multiple comparisons
☐	☒	A full description of the statistical parameters including central tendency (e.g. means) or other basic estimates (e.g. regression coefficient) AND variation (e.g. standard deviation) or associated estimates of uncertainty (e.g. confidence intervals)
☒	☐	For null hypothesis testing, the test statistic (e.g. *F*, *t*, *r*) with confidence intervals, effect sizes, degrees of freedom and *P* value noted<br>*Give P values as exact values whenever suitable.*
☒	☐	For Bayesian analysis, information on the choice of priors and Markov chain Monte Carlo settings
☒	☐	For hierarchical and complex designs, identification of the appropriate level for tests and full reporting of outcomes
☒	☐	Estimates of effect sizes (e.g. Cohen's *d*, Pearson's *r*), indicating how they were calculated

Our web collection on statistics for biologists contains articles on many of the points above.

Software and code

Policy information about availability of computer code

Data collection	SoftMax Pro 6.2.2. was used for luminescence, fluorescence and absorbance data acquisition. LabSolutions 5.71 was used HPCL data acquisition. Topspin 3.5 (Bruker Biospin) and IconNMR ver. 4.2 (Bruker Biospin) for acquisition of NMR data. Huawei Mate 30 pro cell phone inbuilt software (android 10) was used for photographic data acquisition. Image Lab 5.2.1 was used for blot image acquisition. AnalySIS docu 5.0 was used for microscope image acquisition.
Data analysis	MS Excel was used for biosensor data analysis and processing. Origin pro 2020 9.7.0.188 was used for curve fitting of dose-response curve data. ChemDraw Professional 19.1 for chemical structures and MS Powerpoint for the preparation of illustrations. Topspin 3.5 was used for processing of NMR data. RawTherapee 5.8 and ImageJ 1.53C (with the Fiji plugin suite) were used for picture color analysis.

For manuscripts utilizing custom algorithms or software that are central to the research but not yet described in published literature, software must be made available to editors and reviewers. We strongly encourage code deposition in a community repository (e.g. GitHub). See the Nature Portfolio guidelines for submitting code & software for further information.

Data

Policy information about availability of data

All manuscripts must include a data availability statement. This statement should provide the following information, where applicable:

- Accession codes, unique identifiers, or web links for publicly available datasets
- A description of any restrictions on data availability
- For clinical datasets or third party data, please ensure that the statement adheres to our policy

Data supporting the findings of this work are available within the paper and its Supplementary Information files. A reporting summary for this Article is available as a Supplementary Information file. The datasets generated and analyzed during the current study are available from the corresponding author upon request. The

source data underlying Tables in the Supplementary informationv file will be provided as a Source Data file prior to publication.

Field-specific reporting

Please select the one below that is the best fit for your research. If you are not sure, read the appropriate sections before making your selection.

☒ Life sciences ☐ Behavioural & social sciences ☐ Ecological, evolutionary & environmental sciences

For a reference copy of the document with all sections, see nature.com/documents/nr-reporting-summary-flat.pdf

Life sciences study design

All studies must disclose on these points even when the disclosure is negative.

Sample size	Predetermination of sample size was not used in this work. Please refer to the section "replication" below for how samples were allocated and analyzed.
Data exclusions	No data exclusion.
Replication	Unless otherwise mentioned, all measurements were done from at least three biological replicates as pilot experiments suggested sufficient effect size. This the usual practice in the field and we consider this sample size adequate to extract valid conclusions. All attempts at replication of the experiments were successful.
Randomization	20 96-well plates containing compounds from the University of Copenhagen "Chemical biology and HTS" facility compound library were randomly chosen for high-throughput screening experiments. A pipetting robot was used to distribute the contents of 4 randomly chosen plates into one of 5 384 well plates. Each of the 384 well plates was assayed in the original (random) order. Otherwise, randomization was not relevant for this work.
Blinding	Blinding was not used in this work because most experiments produced outcomes that could be batch analyzed giving no opportunity for experimenter bias to be introduced.

Reporting for specific materials, systems and methods

We require information from authors about some types of materials, experimental systems and methods used in many studies. Here, indicate whether each material, system or method listed is relevant to your study. If you are not sure if a list item applies to your research, read the appropriate section before selecting a response.

Materials & experimental systems

n/a	Involved in the study	
☐	☒	Antibodies
☒	☐	Eukaryotic cell lines
☒	☐	Palaeontology and archaeology
☒	☐	Animals and other organisms
☒	☐	Human research participants
☒	☐	Clinical data
☒	☐	Dual use research of concern

Methods

n/a	Involved in the study	
☒	☐	ChIP-seq
☒	☐	Flow cytometry
☒	☐	MRI-based neuroimaging

Antibodies

Antibodies used	Primary antibody (#3724 anti HA-Tag (C29F4) Rabbit mAb, lot 10, Cell Signaling), secondary antibody (Polyclonal Swine Anti-Rabbit Immunoglobulins/HRP, P021702-2 DAKO)
Validation	Primary antibody (anti HA-Tag (C29F4) validated in S. cerevisiae for Western Blot (Wang, et al., 2015 Nature 524(7566):481-4)
